# Factors That Impact Acceptance of COVID-19 Vaccination in Different Community-Dwelling Populations in China

**DOI:** 10.3390/vaccines10010091

**Published:** 2022-01-08

**Authors:** Jinhua Pan, Kezhong A, Zhixi Liu, Peng Zhang, Zhiyin Xu, Xiaoqin Guo, Guangtao Liu, Ao Xu, Jing Wang, Xinyu Wang, Weibing Wang

**Affiliations:** 1 Key Lab of Public Health Safety of the Ministry of Education, School of Public Health, Fudan University, Shanghai 200032, China; 19111020003@fudan.edu.cn (J.P.); 18211020073@fudan.edu.cn (Z.L.); 20211020012@fudan.edu.cn (A.X.); 17301020092@fudan.edu.cn (X.W.); 2 Department of Immunization Programme Institute, Qing Hai Center for Disease Prevention and Control, Qinghai 810007, China; akz1307@126.com; 3 Huzhou Center for Disease Prevention and Control, Huzhou 313000, China; hzjkzp@163.com (P.Z.); guangtl@126.com (G.L.); 4 Department of Infections Disease Prevention and Control, Minhang Center for Disease Prevention and Control, Shanghai 201100, China; fangyi_mh@163.com; 5 Songjiang Center for Disease Prevention and Control, Shanghai 201600, China; guoxiaoqin1102@163.com; 6 Department of Health and Environmental Sciences, Xi’an Jiaotong-Liverpool University, Suzhou 215000, China; Jing.Wang18@student.xjtlu.edu.cn

**Keywords:** COVID-19, vaccine acceptance rate, influencing factors, multi-level logistic regression

## Abstract

(1) Background: It is important to improve vaccination strategies and immunization programs to achieve herd immunity to infectious diseases. (2) Methods: To assess the acceptance of COVID-19 vaccination, we conducted face-to-face surveys and online surveys in Shanghai, Zhejiang, and Qinghai provinces. A fixed-effect model and a random effects model were used to analyze factors associated with the acceptance of COVID-19 vaccination. (3) Findings: We initially recruited 3173 participants, 3172 participants completed the full questionnaire (the response rate was nearly 100%), of which 2169 were valid questionnaires, with an effective rate of 87.3%. The results indicated that 82.6% of participants were willing to receive vaccination when it was available in the community, and 57.2% of deliverymen, 43.3% of medical workers, 78.2% of parents of primary and secondary school children, and 72.2% of parents of preschool children were willing to receive vaccination. The models showed that participants who were male (female vs. male: OR = 1.49, 95% CI (1.12, 1.98)), 60 to 69 years-old (60–69 vs. <30: OR = 0.52, 95% CI (0.29, 0.92)), had less education (medium vs. low: OR = 1.50, 95% CI (1.05, 2.23)), had good health status (good vs. low: OR = 0.36, 95% CI (0.15, 0.88)), and had positive attitudes and trust (OR = 0.14, 95% CI (0.10, 0.20)) in vaccines approved by the National Health Commission were more likely to accept vaccination. Participants also had an increased vaccination acceptance if it was recommended by government sources, doctors, relatives, or friends. Most participants learned about COVID-19 vaccination from television, radio, and newspapers, followed by community or hospital campaigns and the internet. (4) Conclusions: Government sources and doctors could increase the acceptance of vaccination by promoting the efficacy and safety of COVID-19 vaccination by the use of mass media and emphasizing the necessity of vaccination for everyone.

## 1. Introduction

The current 2019 coronavirus disease (COVID-19) pandemic, caused by the severe acute respiratory syndrome coronavirus 2 (SARS-CoV-2), accounts for a significant amount of the total global disease burden [1,2]. As of 14 May 2021, there were 161,367,221 confirmed cases worldwide, and 103,938 cases in China. To slow the spread of infection and mitigate the adverse health effects, public health administrations in many countries implemented various control measures, such as quarantines, partial and comprehensive lockdowns, the closing of schools and businesses, and promoting mask-wearing in public areas [3]. Although these measures were helpful, resurgences of COVID-19 were reported in many regions after the resumption of normal activities [4,5,6]; thus, indicating an urgent need for effective long-term preventive measures. COVID-19 vaccination is generally considered the most effective method to control the spread of this disease [4]. At present, more than ten COVID-19 vaccines have been approved for marketing in different countries [7], and numerous countries have successfully implemented vaccination programs, including China and the United States. Although many countries have achieved significant progress, two major challenges must be overcome: the poor public acceptance of vaccination and the implementation of interventions that increase the public acceptance of vaccination [8].

Many previous studies have examined the public acceptance of COVID-19 vaccination. For example, Reiter et al. [9] stated that 69% of participants in the United States were willing to receive COVID-19 vaccination. Harapan et al. [10] found that 93.3% of individuals in Indonesia were willing to receive a vaccine that had a 95% effectiveness, but only 67.0% were willing if the vaccine had a 50% effectiveness. Lazarus et al. [11] reported that people from China had the highest acceptance of COVID-19 vaccination (88.6%), and Poland had the lowest acceptance (27.3%). However, these studies were all conducted online, and, thus, people without internet access were excluded. Moreover, because almost all of these studies were web-based, this may have compromised the quality of the survey and led to population biases.

However, the real COVID-19 vaccination uptake rate could be much lower than the acceptance after introducing the vaccine and promoting mass immunization programs [8]. The identification of factors related to the acceptance or rejection of COVID-19 vaccination is essential for the design of strategies that improve vaccine coverage in general populations. Some previous studies found that positive attitudes about COVID-19 vaccination were associated with learning about vaccination from government sources, male sex, educational attainment, increased frequency of flu vaccination, and having health insurance [8,12]. However, these were online surveys that used a fixed-effect model for the analysis, and did not consider the effect of geography among participants.

Our general purpose is to provide a more comprehensive understanding of the public acceptance of COVID-19 vaccination and the factors that influence its acceptance. Thus, we performed face-to-face interviews to assess vaccine acceptance in general community-dwelling populations and a larger online survey to assess the attitudes of healthcare workers, deliveryman, the parents of preschoolers, and the parents of primary and secondary school students. We then used a fixed-effect model and a random effect model to analyze the data. This approach is critical for the development of improved strategies that promote the acceptance of COVID-19 vaccination, particularly the vaccination of individuals living in the general community. A comparison of the results of this study with those of previous studies may help to identify changes in the acceptance of vaccination at different times and in different populations.

## 2. Materials and Methods

### 2.1. Ethics Statement

The Ethics Committee of the School of Public Health of Fudan University approved this study (approval no: IRB#2020-11-0758) and all participants or their guardian provided written informed consent.

### 2.2. Study Sites

**Figure 1:**
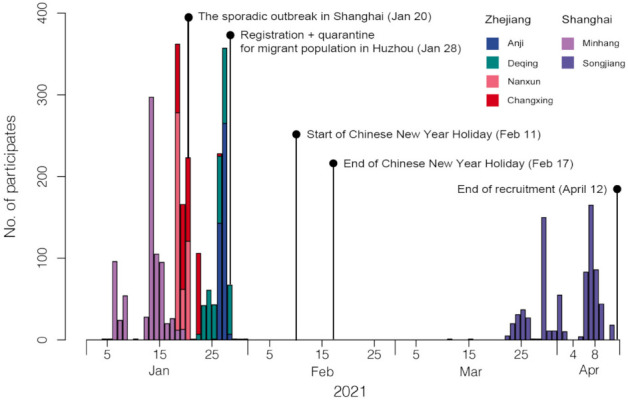
Timeline of the survey. The recruitment date from 4 January 2021 to 12 April 2021. The investigation was suspended for a period of time because of the sporadic outbreak in Shanghai, the Chinese New Year holidays, and the implementation of quarantine policies during the Spring Festival travel rush.

Between December 2020 and April 2021, face-to-face surveys targeting the general population were performed in Shanghai and Huzhou (Figure 1), cities that have very different characteristics. Shanghai is a wealthy and highly connected international hub with a high population density and has experienced continuous importations of COVID-19 cases. Huzhou is a middle-sized city that has a more modest average income and a lower population density. These face-to-face interviews were conducted in two districts of Shanghai (Songjiang and Minhang districts) and in four districts of Zhejiang (Nanxun, Anji, Deqing, and Changxing). Online surveys were also conducted in Shanghai, Huzhou, and Qinghai (a remote region with a low population density and a low average income).

### 2.3. Sampling

For the face-to-face survey, 120 neighborhoods were sampled using multi-stage stratified sampling that was proportional to the population size (Supplementary Materials S1: Figure S1). Then, a convenience sample of 25 households per neighborhood was selected as broadly representative of the geographic characteristics of the whole neighborhood population (Supplementary Materials S1: Figure S2). One person from each household was invited to participate until there were 2966 participants with appropriate age and gender distribution (sampling design and study population, Supplementary Materials S1: Supplementary Text S1). Individuals of all ages who were living in the selected neighborhoods more than 2 weeks and did not intend to move from these districts during the following 2 weeks were eligible for inclusion.

For the online survey, four key populations were selected from Shanghai, Huzhou, and Qinghai, so that each level of hospital (primary, secondary, tertiary) was considered (sampling design and study population, Supplementary Materials S1: Supplementary Text S1). Healthcare workers, cold-chain transportation workers, takeaway workers, and couriers were selected as the key populations. Two to four kindergartens were selected as clusters within each region using simple random sampling (SRS, Supplementary Materials S1: Supplementary Text S1 and Supplementary Materials S2: Supplementary Text S2 for sampling and school details) and two to four senior high schools, junior–middle schools, and primary schools were also selected in each region (Supplementary Materials S1: Supplementary Text S1 and Supplementary Materials S2: Supplementary Text S2 for sampling and school details). One class from each grade was selected in each senior high, junior–middle school, and primary school. Each online survey participant voluntarily signed an informed consent agreement. The target sample size was 1483 for each category. (Supplementary Materials S1: Supplementary Text S1 for population details).

### 2.4. Data Collection

#### 2.4.1. Face-to-Face Survey

After signing the informed consent document, each participant received a questionnaire that was developed based on previous research [10,13,14,15,16,17,18,19,20] and guidance of experts from Fudan University, Zhejiang University, and Duke University. A direct face-to-face interview was performed by investigators who received the same training (Supplementary Materials S1: Supplementary Text S1 for quality control).

The questionnaire collected information on demographics (region of residence, age, sex, level of education, income, basic diseases, and occupation), knowledge, attitudes, and acceptance of COVID-19 vaccination, and willingness to pay for vaccination. Participants were asked the following general question: ‘If a COVID-19 vaccine was free and available to you, would you take it?’, and were then asked to register their level of agreement to the following response: ‘I would follow doctor’s, government’s, relatives’, and friends’ recommendation to receive a COVID-19 vaccine once the government has approved it as safe and effective’. The level of agreement to this response was rated using a six-point Likert scale (‘completely possible’, ‘somewhat possible’, ‘neutral/no opinion’, ‘somewhat impossible’, ‘completely impossible’, and “I do not know”.

Each participant’s knowledge of COVID-19 vaccination and their reasons for accepting vaccination were also examined. The attitudes about two COVID-19 vaccines were determined: the domestic vaccine (approximately 80% effective) and the imported vaccine (approximately 95% effective). The acceptance and willingness of the participant to pay for vaccination of himself/herself, his/her children, and elderly family members (≤60 years old) were assessed for each vaccine. Information about whether the participant or a family member was sickened or died from COVID-19 was on the ‘front lines’ during implementation of pandemic control and treatment measures, was in quarantine because of contact history, the distance from the participant’s home to the nearest vaccination site, and the number of years the participant lived in the sampled area were recorded.

The responses to different questions were determined for different groups defined by age (<30, 30–39, 40–49, 50–59, 60–69, and >70 years old), gender (male, female), education level (less than high school (low), high school or technical secondary school (medium), bachelor’s degree or above (high)), marital status (married, other (single, divorced, or widowed)), and employment status (employed, unemployed (full-time housewife, retired, and unemployed)). In addition, questions were used to assess knowledge (4 questions), attitude (2 questions), and behavior (5 questions) related to COVID-19 vaccination (questions about knowledge, attitude and behavior details can be seen in Supplementary Materials S1: Supplementary Text S1). A correct answer was scored as ‘1’, an incorrect answer as ‘0’, and total scores were determined for knowledge, attitude, and behavior.

#### 2.4.2. Online Survey

After signing the informed consent agreement, participants in the online survey completed the questionnaire online. This questionnaire was simpler than the face-to-face questionnaire, and collected information demographic information (age, sex, level of education, income, and occupation). The general vaccine-related question was, ‘If a COVID-19 vaccine was free and available to you, would you take it?’.

#### 2.4.3. Statistical Analysis

Categorical variables were expressed as proportions and continuous variables as means (standard errors). Multilevel logistic regression was used for analysis. Simple logistic regression was used for sensitivity analysis. To better explore the influencing factors in the binary data, we explored two models: (i) a fixed-effect logit mode, simple logistic regression, (ii) a mixed-effect logit model (generalized linear mixed-effect model, GLMM, for binary data with logit link). We reported the results of the multilevel logistic regression model if the two models had different results as it represented the preferable choice given the structure of the analyzed data. An outcome was defined as ‘1’ for an answer of ‘completely agree’ or ‘somewhat agree’, and ‘0’ for any other response (statistical analysis, Supplementary Materials S1: Supplementary Text S1). For missing data, a random forest model was used for imputation (mice package and randomForest package in R). Bootstrapping was used to estimate 95% confidential intervals of vaccine acceptance. All data analyses were conducted using R version 3.6.0.

## 3. Results

### 3.1. Face-to-Face Survey

#### 3.1.1. Demographic Characteristics

We initially recruited 3173 participants, 3172 participants completed the full questionnaire (the response rate was nearly 100%), of which 2169 were valid questionnaires (did not receive COVID-19 vaccination and within the sampling area), with an effective rate of 87.3% (Figure S3). Among these 2769 participants, 1286 (46.6%) were female, 1469 (53.0%) were from rural regions and 1300 (46.9%) were from urban areas (Table 1 and Table S6), and 25.7% had incomes of more than 50 to 100 thousand CNY per year. More than half of the respondents (53.2%) had a ‘low level’ of education (completion of junior high school or less). The mean age was 45.95 years, and there were similar numbers of participants in the different age groups (Supplementary Materials S1: Figure S4).

#### 3.1.2. Vaccine Acceptance in the Community

**Figure 2:**
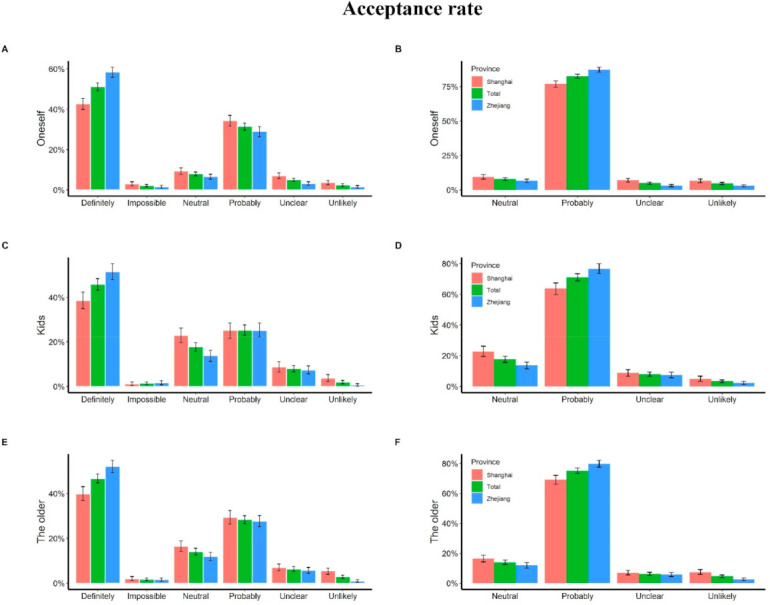
Vaccine acceptance of people in the community. (**A**,**B**) The acceptance rate of participants reported: (**C**,**D**) the willingness to vaccinate their kids; (**E**,**F**) the willingness to vaccinate the older in their family. For panel (**B**,**D**,**F**), we combined the options “definitely” and “probably” into probably, and combined “impossible” and “unlikely” into unlikely. For each plot, the read bar represents the acceptance rate of Shanghai, the blue bar represents the acceptance rate of Zhejiang, and the green bar represents the overall acceptance rat, the tentacles on the bars represent averaged results of bootstrap simulations with 95% confidence interval.

Our analysis of participants in the face-to-face survey indicated that 2286 (82.6%) of them would accept vaccination if a ‘free and effective’ COVID-19 vaccine was available (Figure 2). There were more positive responses in Zhejiang (1298/1486, 87.3%, *p* < 0.0001) than in Shanghai (988/1283, 77.0%).

Of the 1418 participants who reported they had children under the age of 18, 649 (45.8%) stated they will definitely choose to have their children vaccinated (238 (38.4%) in Shanghai, 411 (51.4%) in Huzhou), 357 (25.2%) stated they will probably choose to have their children vaccinated (156 (25.2%) in Shanghai, 201 (25.2%) in Huzhou), while 20 (1.4%) and 28 (2.0%) parents stated it is impossible and unlikely to vaccinate their children in the future, respectively (Figure 2).

Among the 2169 participants who reported their family having members above 60 years old, 1012 (46.7%) stated they will definitely choose to vaccinate their elderly family members (381 (39.8%) from Shanghai and 631 (52.1%) from Huzhou), 616 (28.4%) stated they will probably choose to vaccinate their elderly family members (280 (29.3%) were from Shanghai, 336 (27.7%) were from Huzhou), and 64 (3.0%) were unlikely to vaccinate their elderly family members. In total, 38 (1.8%) respondents stated it is impossible to vaccinate the elderly in their families (Figure 2).

#### 3.1.3. Methods to Improve Acceptance of Vaccination

**Figure 3:**
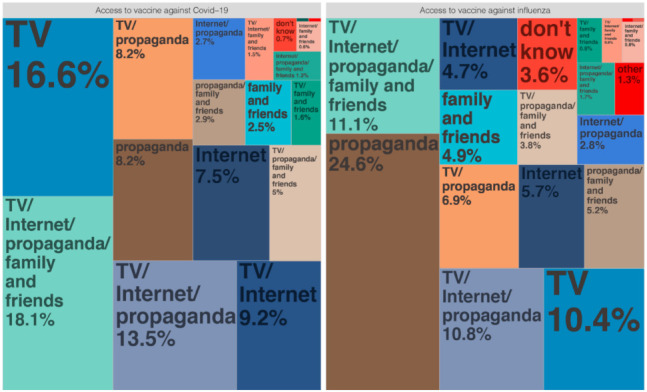
Sources of different vaccine information. TV means TV, radio, and newspapers; internet included QQ, WeChat, Weibo, news websites, etc.; propaganda means community or hospital propaganda.

**Figure 4:**
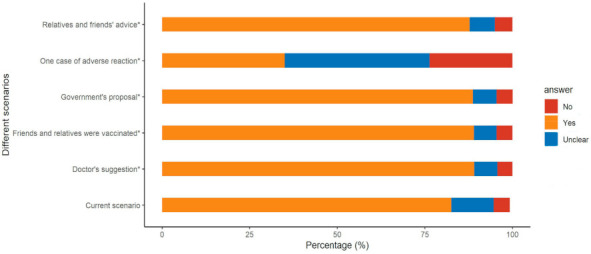
Acceptance under different scenarios. * Had statistical significance (compared with current scenario).

We assessed the sources of each participant’s knowledge about influenza vaccination in the face-to-face survey. Among the 2769 participants, 93 (4.2%) knew little about the flu vaccine; 1465 (66%) learned about the flu vaccine from publicity provided by the local community or hospital; 1007 from newspapers, videos, or television; 810 from the internet (QQ, WeChat, Weibo, news websites, etc.); 507 from family and friends; 36 from other sources. In contrast, knowledge about COVID-19 vaccination was mainly from television, videos, and newspapers (1619, 58.5%), followed by community or hospital campaigns (1270, 45.9%), the internet (1188, 42.9%), family and friends (723), and other sources (2). Sixteen participants stated they knew nothing about COVID-19 vaccination (Figure 3). The vaccination acceptance rate was greater for those who learned about it from government publicity and suggestions by a doctor, relative, or friend, but was less for those who were worried about vaccine safety (Figure 4).

**Figure 5:**
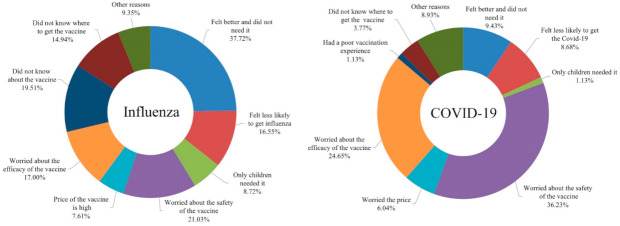
Reasons for not wanting to vaccinate.

Among the 2235 (80.7%) participants who did not receive the influenza vaccine, 843 (37.72%) stated they were in good health and did not need it, 370 (16.55%) stated they were unlikely to contract influenza, 195 (8.72%) believed that only children needed the vaccine, 470 (21.03%) worried about vaccine safety, 170 (7.61%) reported it was too expensive, 380 (17.00%) worried about the efficacy, 436 (19.51%) never heard about the vaccine, and 334 (14.94%) did not know where to receive the vaccine (Figure 5). Thus, the major reason participants did not receive the influenza vaccine was that they thought it was unnecessary because they already had a good health status and concerns about the vaccine safety (Figure 5).

Among the 483 participants (17.44%) who refused or were hesitant about the COVID-19 vaccination, most were worried about the safety (288, 59.63%) and efficacy (196, 40.58%). Additionally, 75 (15.53%) participants thought they were in good health and did not need the vaccine, 69 (14.29%) believed they had a low risk of infection, 48 (9.94%) were worried about the high price, 30 (6.21%) did not know where to receive the vaccine, 9 previously had poor vaccination experiences, and 71 (14.70%) had other reasons, such as old age, presence of a chronic disease, and other factors (Figure 5).

The simple logistic (AIC = 1628.2) and multilevel logistic (AIC = 1625.8) models indicated that males were more likely to accept COVID-19 vaccination than females (Table 2). In addition, those with a ‘low level’ of education were more likely to accept the COVID-19 vaccine than those with a ‘medium level’ of educational. Relative to participants with poor health, those with good health were less likely have negative views of COVID-19 vaccination (simple logistic model: OR = 0.35, 95 CI% (0.15, 0.88); multilevel logistic model: OR = 0.36, 95 CI% (0.15, 0.88)). Higher attitude and behavioral scores were also associated positively with vaccine acceptance. Those who did not know whether to choose an imported or domestic vaccine were less likely to accept vaccination than those who preferred the imported vaccine. The logistic model indicated that participants from rural areas were more likely to accept vaccination than those from urban areas (OR = 0.59, 95% CI (0.44, 0.79)). The multilevel logistic model indicated that participants aged 60 to 69 years were more likely to accept vaccination than those less than 30 years old (OR = 0.52, 95% CI (0.29, 0.92)) (Table 2).

### 3.2. Online Survey

#### 3.2.1. Demographic Characteristics

We also conducted an online survey in Shanghai, Qinghai, and Huzhou to determine the acceptance of vaccination by delivery workers, healthcare workers, parents of preschoolers, and parents of primary and secondary school students (*n* = 20,595). Among these participants, 2879 were delivery workers, 4852 were parents of preschoolers, 3826 were healthcare workers, and 9038 were parents of primary and secondary school students. A total of 17,206 of these individuals (1335 delivery workers, 2001 healthcare workers, 4847 parents of preschoolers, and 9017 parents of primary and secondary school students) completed the full questionnaire and passed the audit based on verification questions (Supplementary Materials S1: Figure S3), which included naming the capital of China, work unit, birth date, and geographic location (verified by comparison with the internet protocol address).

Most of the delivery workers were male (85.6%), more than half of them had a ‘low level’ of education (50.2%), and 40.8% had incomes below 10,000 CNY in 2019. Most of the healthcare workers were female (76.9%), more than half of them had a ‘high level’ of education (90%), and 27.7% had incomes of 30,000 to 100,000 CNY in 2019. Among the parents of preschoolers, 52.4% were male and 22.6% had incomes of 500,000 to 1,000,000 CNY in 2019. Among the parents of primary and secondary school students, 51.9% were male and 22.6% had incomes of 100,000 to 300,000 Chinese CNY in 2019 (Table 1 and Table S1).

#### 3.2.2. Vaccine Acceptance in the Four Key Populations

**Figure 6:**
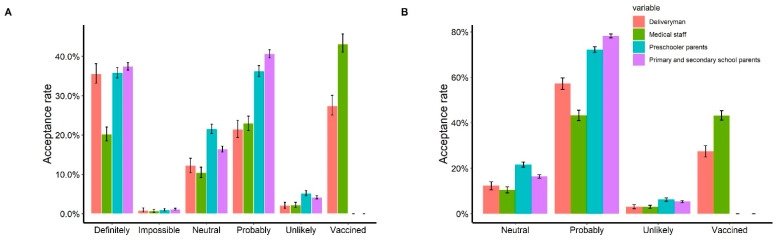
Vaccine acceptance of four key populations. (**A**) The acceptance rate of four key populations; (**B**) the acceptance rate of four key populations. We combined the options “definitely” and “probably” into probably, and combined “impossible” and “unlikely” into unlikely. For each plot, the read bar represents the acceptance rate of deliveryman, the green bar represents the acceptance rate of medical staff, the blue bar represents the acceptance rate of preschoolers’ parents, and the purple bar represents the acceptance rate of primary and secondary school students’ parents; the tentacles on the bars represent averaged results of bootstrap simulations with 95% confidence interval.

Among the four key groups in the online survey, 57.2% of delivery workers, 43.3% of healthcare workers, 78.2% of the parents of primary and secondary school students, and 72.2% of the parents of preschool children indicated they would accept vaccination (Figure 6). Notably, the parents of primary and secondary school students had the most positive responses (7052/9017, 78.2%), and healthcare workers had the fewest positive responses (867/2001, 43.3%). We also explored the acceptance rate under two different hypotheticals vaccination scenarios that considered the vaccine’s origin and efficacy, and the probability of adverse reactions (results, Supplementary Materials S1: Supplementary Text S1).

## 4. Discussion

We investigated the acceptance of COVID-19 vaccination and identified factors related to this acceptance using an online survey and a face-to-face survey of different community-dwelling populations in China. The results indicated that acceptance varied among different groups within a population and also among different populations (we used the results of the face-to-face survey to obtain the acceptance rate of COVID-19 in the community and identified factors related to this acceptance; we just used the results of the online survey to collect the acceptance of COVID-19 vaccination among the four key populations).

### 4.1. Face-to-Face Survey

In particular, a comparison of the general community populations indicated a greater acceptance of vaccination in Zhejiang (87.3%) than in Shanghai (77.0%). Our results indicated a strong desire for vaccination in the general community, suggesting support for large-scale vaccination programs. The administration of hundreds of millions of vaccines may present a significant logistical challenge to the government and policymakers in China. Therefore, we suggest there should be a focus on the establishment of vaccination sites and the rapid allocation of vaccination resources and other preparations to prevent shortages of vaccines so that a rapid population immunity can be established.

The identification of factors that influence the acceptance of COVID-19 vaccination—both barriers and facilitators—is important for the design of effective strategies that improve vaccine coverage in the general community. We found that males had a stronger acceptance of vaccination than females, possibly because previous studies reported greater risks of complications and death due to COVID-19 in males than females [21]. Similarly, we also observed a greater acceptance of vaccination in people aged 60 to 69 years than those under 30 years old, possibly because there are greater risks of complications and death due to COVID-19 in the elderly [12]. These results suggest that many people may misunderstand that having a somewhat lower risk of complications or death from COVID-19 means that they can ignore the need for vaccination. Therefore, future vaccination campaigns should guide the entire public to understand the importance of vaccination and universal coverage for an establishing population-wide immunity, and that it is necessary to include individuals who are not in high-risk groups. In the face of the COVID-19 pandemic, it is unrealistic to maintain a prolonged lockdown strategy, because this will lead to a significant economic burden and other problems. We found that people with more education and those from urban areas were less likely to accept vaccination, suggesting the need to focus on these groups to improve vaccine coverage, which was consistent with the previous studies [11].

Our survey confirmed that general community-dwelling populations had greater trust in COVID-19 vaccines that were approved by the National Health Commission and believed that vaccination was an effective protective measure. Furthermore, participants who were concerned about their own health and kept a positive attitude were more likely to accept vaccination. This could be because the Chinese government and medical workers outwardly expressed confidence, reliability, and credibility in their promotions of vaccination. Thus, within a year since the onset of the COVID-19 pandemic, there has been an increased awareness of the need for self-protection (wearing masks, frequent hand washing, maintaining social distance, etc.) and a decreased use of public transportation. These measures also helped to decrease the incidence of other respiratory infectious diseases. We found that people with a strong sense of self-protection were more likely to comply with prevention and control recommendations from the government.

In addition to building public trust, reliable sources of information and guidance are crucial for vaccine mobilization and disease control [22]. Our results indicated that, in contrast to previous vaccines, the public acquired most of its information about COVID-19 vaccination from television, radio, newspapers, hospital publicity, and the internet. Moreover, recommendations from the government, doctors, friends, and relatives increased the acceptance of vaccination; the government and doctors had the greatest impact, and vaccine information from these two sources increased the acceptance of vaccination to nearly 90%.

### 4.2. Online Survey

A comparison of different groups within populations indicated that acceptance was greatest among parents of primary and secondary school students (78.2%), and lowest among medical workers (43.3%). The low vaccination rate of medical workers may be due to the fact that some medical workers had been vaccinated at the time of our survey. By adding up the vaccinated people and the unvaccinated people who were willing to be vaccinated, the vaccination rate of medical workers was calculated to be 86.8%. Similarly, at the time of the survey, some of the delivery workers had been vaccinated. We added up those who had been vaccinated to calculate the final vaccination rate of the delivery workers, which amounted to 84.6%. In contrast to previous studies [23,24], we investigated the willingness to be vaccinated in different groups of people. Given that many people living in the community do not use mobile phones, our combined use of a face-to-face and online survey provided a more reliable assessment of vaccine acceptance in the general community and a stronger scientific basis for developing vaccination policies for general communities.

This study had some strengths, such as a large sample size, the combination of an online and offline survey, combination of general population and several special population surveys, and a reasonable sampling method. Importantly, the acceptance of vaccination in a population is likely to change over time. The results of this study reported herein could be compared with previous and future studies. This study had some limitations. In particular, this being a cross-sectional survey, it may have produced a bias, such as recall bias and selection bias. About the recall bias, we tried to minimize the number of questions that required recall and just collected information such as the basic characteristics, willingness to vaccinate, and attitudes toward the COVID-19 vaccine in the questionnaire. In order to reduce the selection bias, we wrote the research plan in advance at the beginning of the study, including a strict sample selection. During the implementation of the survey, the sampling principle remained unchanged. For the respondents with no response bias, only one of the respondents in this study did not complete the whole questionnaire; therefore, we could neglect the correspondence bias in this study.

## 5. Conclusions

At present, the COVID-19 epidemic in China is mostly under control and there is a widespread acceptance of vaccination, especially in community-dwelling individuals. We found that men, rural residents, and those with less education were more likely to accept vaccination. Governments have to introduce new strategies to persuade females, those with more education, and urban residents to accept vaccination. Among individuals who were vaccine refusers or hesitators, most were worried about vaccine safety and efficacy. Most people obtained knowledge about COVID-19 vaccination from television, videos, and newspapers, followed by community or hospital campaigns, and then the internet. Recommendations from the government, doctors, friends, and relatives increased the acceptance of vaccination. Therefore, we suggest that increasing the success of mass vaccination campaigns and public acceptance of vaccination, especially for females, urban residents and those with more education, can be achieved if governments and doctors promote the efficacy and safety of vaccines using television, radio, and the internet to emphasize the importance of universal vaccination.

## Figures and Tables

**Table 1:** Description of the participants.

	Community Residents	Deliverymen	Health Workers	Parents of Preschoolers	Parents of School Students *
	*n* = 2769	*n* = 1335	*n* = 2001	*n* = 4847	*n* = 9017
Age (mean (SD))	45.95 (19.29)	31.52 (7.45)	34.70 (9.46)	4.61 (1.51)	11.21 (3.55)
Province (%)					
Zhejiang	1283 (46.3)	317 (23.7)	534 (26.7)	2127 (43.9)	1206 (13.4)
Qinghai	1486 (53.7)	68 (5.1)	779 (38.9)	1996 (41.2)	7251 (80.4)
Shanghai		950 (71.2)	688 (34.4)	724 (14.9)	560 (6.2)
Sex (%)					
Female	1483 (53.6)	232 (17.3)	1539 (76.9)	2305 (47.5)	4341 (48.1)
Education (%)					
Low	1390 (50.2)	520 (39.0)	35 (1.7)	N/A	N/A
Median	542 (19.6)	558 (41.8)	156 (7.8)	N/A	N/A
High	755 (27.3)	257 (19.0)	1810 (90.5)	N/A	N/A
Income (thousand CNY, %)					
<10	574 (20.7)	545 (40.8)	376 (18.8)	959 (19.8)	3490 (38.7)
10–30	587 (21.2)	192 (14.4)	277 (13.8)	759 (15.7)	2065 (22.9)
30–50	648 (23.4)	239 (17.9)	266 (13.3)	746 (15.4)	1472 (16.3)
50–100	711 (25.7)	288 (21.6)	544 (27.2)	1096 (22.6)	1165 (12.9)
100–200	195 (7.0)	59 (4.4)	456 (22.8)	781 (16.1)	530 (5.9)
>200	54 (2.0)	12 (0.9)	82 (4.1)	506 (10.4)	295 (3.3)

* School children: students in primary and secondary schools.

**Table 2:** Impact factors associated with COVID-19 vaccine acceptance.

	Univariate Logistic Model		Multivariate Logistic Model	
	Adjusted OR (95% CI)	Crude OR (95% CI)	Adjusted OR (95% CI)	Crude OR (95% CI)
Sex: female vs. male	1.48 (1.12, 1.96) ^†^	1.56 (1.28, 1.91) ^†^	1.49 (1.12, 1.98) ^†^	1.55 (1.27, 1.90) ^†^
Age				
30–39 vs. <30	0.77 (0.48, 1.23)	0.84 (0.61, 1.16)	0.76 (0.47, 1.22)	0.86 (0.62, 1.18)
40–49 vs. <30	0.82 (0.49, 1.35)	0.72 (0.51, 1.00)	0.78 (0.47, 1.30)	0.80 (0.57, 1.12)
50–59 vs. <30	0.88 (0.53, 1.48)	0.60 (0.43, 0.84) ^†^	0.83 (0.49, 1.40)	0.66 (0.47, 0.92) ^†^
60–69 vs. <30	0.58 (0.32, 1.02)	0.78 (0.56, 1.08)	0.52 (0.29, 0.92) *	0.75 (0.54, 1.05)
>69 vs. <30	0.66 (0.38, 1.15)	0.78 (0.57, 1.05)	0.63 (0.36, 1.11)	0.78 (0.58, 1.06)
Region: rural vs. urban	0.59 (0.44, 0.79) ^†^	0.57 (0.47, 0.70) ^†^	0.87 (0.58, 1.3)	0.91 (0.67, 1.25)
Overseas experience Last month	15.6 (2.51, 127.66) ^†^	2.16 (0.68, 5.98)	14.66 (2.25, 95.41) ^†^	1.74 (0.60, 5.07)
Education				
Medium vs. low	1.64 (1.13, 2.38) ^†^	1.41 (1.09, 1.8) ^†^	1.5 (1.05, 2.23) *	1.19 (0.92, 1.54)
High vs. low	1.13 (0.74, 1.71)	1.20 (0.95, 1.51)	1.07 (0.7, 1.62)	1.02 (0.80, 1.29)
Marriage: others vs. married	1.19 (0.84, 1.69)	1.50 (1.19, 1.88) ^†^	1.13 (0.79, 1.62)	1.39 (1.10, 1.75) ^†^
Employment: unemployed vs. employed	1.15 (0.81, 1.65)	1.13 (0.91, 1.39)	1.09 (0.76, 1.56)	0.97 (0.78, 1.21)
Quarantined: yes vs. no	0.24 (0.08, 0.58) ^†^	0.81 (0.44, 1.40)	0.25 (0.09, 0.64) ^†^	0.86 (0.48, 1.53)
Health				
Good vs. low	0.35 (0.15, 0.88) *	0.43 (0.24, 0.79) ^†^	0.36 (0.15, 0.88) *	0.46 (0.25, 0.82) ^†^
Medium vs. low	0.42 (0.17, 1.07)	0.53 (0.29, 0.99) *	0.41 (0.16, 1.01)	0.50 (0.27, 0.94) *
Preference of imported or domestic vaccines				
Both fine vs. domestic	1.12 (0.80, 1.55)	1.48 (1.15, 1.9) ^†^	1.09 (0.78, 1.52)	1.43 (1.11, 1.85) ^†^
Imported vs. domestic	1.52 (0.92, 2.43)	1.6 (1.07, 2.34) *	1.5 (0.92, 2.43)	1.71 (1.15, 2.53) ^†^
Unclear vs. domestic	7.52 (4.99, 11.37) ^†^	9.65 (7.05, 13.29) ^†^	7.68 (5.08, 11.61) ^†^	10.22 (7.39, 14.13) ^†^
Behavior	0.74 (0.61, 0.91) ^†^	0.65 (0.57, 0.73) ^†^	0.73 (0.60, 0.90) ^†^	0.65 (0.57, 0.74) ^†^
Attitude	0.14 (0.10, 0.19) ^†^	0.10 (0.08, 0.13) ^†^	0.14 (0.10, 0.20) ^†^	0.10 (0.08, 0.13) ^†^

* *p* < 0.05, ^†^ *p* < 0.01.

## Data Availability

The data presented in this study are available on request from the corresponding author.

## Supplementary Materials

The following are available online at https://www.mdpi.com/article/10.3390/vaccines10010091/s1, Figure S1: Six districts (counties) sampled and 120 neighborhoods locations; Figure S2: Home locations of participants sampled from one neighborhood for instance; Figure S3: Flow chart from enrollment to analysis for the 23,786 participants; Figure S4: Age distribution of the participants in the community; Table S1: Age distribution of Shanghai and Huzhou population vs. our sample in Shanghai and Huzhou; Table S2: Comparison between effective participants in Shanghai and permanent residents in Shanghai; Table S3: Comparison between effective participants in Huzhou and permanent residents in Huzhou; Table S4: Comparison between effective participants in our study and residents in China; Table S5: Acceptance rate of two vaccine scenarios; Table S6: Description of community participants; Table S7: Knowledge, attitude, and practice about COVID-19 and COVID-19 vaccine.
