# Supplementary material for: Factors That Impact Acceptance of COVID-19 Vaccination in Different Community-Dwelling Populations in China
Source: Vaccines (Basel). 2022 Jan 8;10(1):91. doi: 10.3390/vaccines10010091 (PMC8779453; doi:10.3390/vaccines10010091)
Supplement: Supplementary file 1 [file vaccines-10-00091-s001.zip › Supplementary material 1.pdf]

vaccines

Supplementary Text 1

Sampling design and study population

Sampling design

We first sampled two districts in Shanghai and four districts in Huzhou, Zhejiang. Then, we sampled a total of 10 sub-districts from these two districts of Shanghai and from these 10 sub-districts, each of which extracted 6 neighborhoods (the smallest administrative unit in China); and sampled a total of 12 sub-districts from these four districts of Huzhou, and from these 12 sub-districts, each of which extracted 5 neighborhoods, using multi-stage stratified probability proportional to population size (PPS). At the district level, two districts were selected from the total 16 districts of Shanghai by PPS (Songjiang, and Minhang) and four districts were selected from the total five districts of Huzhou by PPS (Nanxun, Changxing, Deqing, and Anji). At the sub-district level, 10 sub-districts in Shanghai within these two districts were sampled by PPS; 12 sub-districts in Huzhou within these four districts were sampled by PPS. The geographical distribution of our sample sites can be seen from Supplementary Fig. S1. At the household level, 60 households per neighborhood were selected with the help of neighborhood committee both in Shanghai and Huzhou. It is hoped that the selected households could be broadly representative of the demographic characteristics of the neighborhood in terms of geographical spread. One person per household was invited to participate in our study until we met our predefined target sizes by age and gender (described below, table S1). A pilot survey was conducted from October 2020 to November 2020 in Xuhui districts, Shanghai.

The key populations were recruited from the aforementioned districts of Shanghai and Zhejiang, as well as the city of Haidong and the city of Haixi in Qinghai province.

We selected Shanghai Mental Health Center, Shanghai Fifth People's Hospital, Renji Hospital, Minhang District Central Hospital, Songjiang District Central Hospital, Songjiang District Fangta Traditional Chinese Medicine Hospital, Songjiang Maternal and Child Health Hospital from Shanghai; Center for Disease Control and Prevention (CDC), Dulan County, Golmud CDC, CDC of Delingha City, CDC of Xunhua County, People's Hospital of Dulan County, People's Hospital of Minhe County and People's Hospital of Golmud City, Traditional Chinese Medicine

Hospital of Delingha City and Traditional Chinese Medicine Hospital of Minhe County from Qinghai Province; Linghu People's Hospital (Nanxun District), Changxing County Hospital of Traditional Chinese Medicine, Anji Third People's Hospital, Nanxun District CDC, Changxing County CDC, Anji County CDC and Deqing County CDC from Huzhou. The surveys were mainly carried out in hospitals of first-level grade-B and above, and CDCs. Sampling methods ensure that each level has hospitals or CDCs selected instead of random sampling. The surveys were carried out in departments that may have come into contact with Covid-19 confirmed patients and their laboratory products, such as Infection Department, Pneumology Department, Fever Clinic, Intensive Care Unit and Laboratory Department. The trained surveyor contacted the directors of the corresponding departments in the selected hospital to explain the detailed rules for filling out the questionnaire. The director of each department was responsible for contacting the medical staff of the relevant departments to fill out the electronic questionnaire. The ratio of doctors to nurses is close to 1:1.

In each of these regions, we selected three types of delivery workers - cold chain transportation workers, takeaways deliveryman and mailman - to participate in the survey. With the help of the person in charge of delivery company, the trained surveyors joined their local work groups, such as a WeChat group, to explain in detailed rules for filling out the questionnaire, and the take-away workers filled out the electronic questionnaire according to the relevant rules and precautions through the shared link.

Two to four kindergartens were selected as clusters within each region using simple random sampling (SRS, Supplementary Text 2 for school details) and random number.

The names of kindergartens in each region are sorted according to the first letter, and those with the same first letter is sorted according to the second letter, so as to assign a random number to each school. Then each school is sorted according to the random number, and the schools with the top random number are selected for inclusion in the study, until the sample size requirements of the region are met. The head teachers of the selected kindergartens shared the electronic questionnaire to the corresponding parent groups, and explained the detailed rules for filling out the questionnaire in the groups, so as to investigate the parents' willingness to have their children vaccinated.

vaccines

Similarly, using the simple random sampling (SRS, Supplementary Text 2 for school details) and random number, we selected an equal number of high schools, middle schools and primary schools in each region. The names of high schools, middle schools and primary schools in each region are sorted according to the first letter, and those with the same first letter is sorted according to the second letter, so as to assign a random number to each school. Then each school is sorted according to the random number, and the schools with the top random number are selected for inclusion in the study, until the sample size requirements of the region are met. We chose one class for each grade in high schools, middle schools and primary schools. The head teachers of the selected class sent the electronic questionnaire to the corresponding parent groups with instructions on how to fill it out in details to investigate the willingness of parents to have their children vaccinated.

Our target sample size was 1,483 for each category of key populations. Individuals of all ages who had lived in the selected districts for longer than two weeks and did not intend to move out of the selected districts in the next two weeks were considered eligible for inclusion in the study.

The sample size allocation at each stage was determined by the sample size calculation shown below.

Sample size calculation

The sample size was calculated based on the key variable - “the acceptability of Covid-19 vaccine”. We assume that intra-class correlations among individuals exist only at the neighborhood level. Comparing our complex sample to the unrestricted sample design, we consider neighborhood-level clustering by multiplying the effective sample size by the design effect. Thus, the sample size is calculated as follows:

$$N=\frac{Z_{\frac{\alpha}{2}}P(1-P)}{\delta^2}*D^2$$

Where the design effect (D^2) denotes the ratio of the variance of a statistic with a complex sample design to the variance of that statistic with a simple random sample or an unrestricted sample of the same size.

Assumptions for sample size calculation:

• 95% confidence, alpha equals 0.05.

• p equals 0.9, referring to a published survey.

• Delta equals 0.05, a specified level of precision.

• D^2 equals 2

Based on these assumptions, a total sample size needed in each category of key populations is 1,186 per province. Assuming that 80% of participants would eventually return a completed questionnaire, we would need to recruit approximately 1,483 participants in each province for the study in each category of key populations.

In this case, we selected 25 households per neighborhood, and thus 3000 households and 120 neighborhoods were needed in total. 6 neighborhoods were sampled per sub-district in Shanghai and 5 neighborhoods were sampled per sub-district in Huzhou, thus a total of 24 sub-districts were required for the two cities. According to stratified probability PPS, we sampled 5 in Minhang, 5 in Songjiang, 3 in Nanxun, 3 in Changxing, 3 in Deqing, and 3 in Anji.

Study population

Six age groups were defined as <30, 30-39, 40-49, 50-59, 60-69, 70 years and above. Table S1 shows the age distribution of the 2010 census population in Shanghai (with a total population of approximately 24.3 million) and Huzhou (with a total population of approximately 2.7 million) compared to our sample, with a final sample size of 3000.

Quality control

Questionnaire Design: After extensive review of relevant domestic and international literatures, the final questionnaire was formed according to the questionnaires from Chinese Center for Disease Control and Prevention (CDC) and the United States Centers for Disease Control and Prevention (CDC) and under the guidance of relevant experts from Fudan University, Zhejiang University and Duke University. The questionnaire survey was conducted strictly in

accordance with the original settings, and cannot be modified at will.

Investigator Training: Under the guidance of experts from the School of Public Health, Fudan University, the relevant provincial and municipal CDCs directly trained the investigators in the investigated areas. The investigators were required to be familiar with the contents and evaluation criteria of the questionnaire, and to carefully review each questionnaire during the field survey.

Pilot Survey: Prior to the formal baseline survey, a pilot survey was conducted in Xuhui District of Shanghai for selected populations, and the questionnaire was modified and improved in time according to the feedback from the pilot survey.

Process: In face-to-face surveys, we used standard terms to inquire about each item in the questionnaire to ensure that there were no omissions or missing items, and we checked the questionnaire, paid the return visit and corrected the problems in time to make sure there were no omissions or logical problems; In online surveys, we set verifiable questions in the middle of the questionnaire, such as the match between IP location and the location selected in the questionnaire, 'where is the capital of China ?' . The received questionnaires were verified every day, the auditors were trained and familiar with the content of the questionnaire.

Data entry: The contents of all questionnaires was proofread and logically analyzed again before data entry. Two investigators carried out double entry. Two other investigators reviewed the input results to ensure the accuracy of the entered data.

Questions about knowledge, attitude and behavior

Four questions about COVID-19 knowledge are demonstrated as follows: 1) Not all people infected with COVID-19 will become severe cases, only the elderly and patients with chronic diseases are more likely to develop severe symptoms. 2) COVID-19 patients cannot transmit the virus to others. 3) Children and young adults do not need to take measures to prevent COVID-19 infection. 4) Isolation and treatment of COVID-19 infection are effective methods to reduce the transmission of the virus. The answer choices are 'right', 'wrong' and 'unclear'. Choosing 'right' scored 1 point, other options scored no points.

The five related questions about behavior are: 1) Whether wearing masks in public places in

the last month. 2) Whether going out as little as possible after the pandemic. 3) Whether reducing the utilization of public transportation after the pandemic. 4) Whether keeping social distance from others after the pandemic. 5) Whether washing hands frequently after the pandemic. The answer choices are 'yes', 'no' and 'uncertain'. Choosing the 'yes' scored 1 point, other options scored no points.

The two attitude-related questions are: 1) Vaccines are very important to protect health. 2) All vaccines marketed in China through the State Food and Drug Administration are safe. The answers choices are 'completely agree', 'agree', 'neutral', 'not quite agree', 'completely disagree' and 'unclear'. Choosing 'completely agree' or 'agree' scored 1 point, 'not quite agree' and 'completely disagree' scored no points, and 'neutral' or 'unclear' scored 0.5 points.

Statistical analysis

The study adopted multilevel logistic regression and logistic regression, and respondents were given a score of 1 if they answered 'completely agree' or 'somewhat agree', and 0 for any other responses. The independent demographic variables included age, gender, employment status, marriage, region, ethnic group, income, basic disease history, education, health status, religion, and their choice of imported or domestic vaccines. We also examined the relationship between the regression outcomes and whether the respondent or someone familiar with the respondent had fought on the front lines of the epidemic; whether the respondent was quarantined because of a history of Covid-19 exposure; as well as the distance from respondent's home to the nearest vaccination site; the length of residence of the respondent lived in the sampling areas; and the level of respondents' attitude, behavior and knowledge about Covid-19 and Covid-19 vaccine, with the calculation of odds ratio (OR), regression coefficient (β) and a 95% confidence interval (CI).

Results

Two scenarios about the vaccine

We also set up two scenarios to ask about the willingness to vaccinate. The first scenario assumes that vaccine A is a domestic vaccine. Its effectiveness is about 80%, and the side effects

are small. The second scenario assumes that vaccine B is an imported vaccine. The effect of this vaccine is about 95% and has less side effects. Among the 2769 subjects surveyed, we found that, overall, the willingness to receive vaccine A is 75.3 (Shanghai: 64.9%, Zhejiang: 84.2%), and the willingness to receive vaccine B is 46.4% (Shanghai: 40.5%, Zhejiang: 51.4%)

Participants' knowledge, practice, and attitudes about Covid-19 and Covid-19 vaccination

Suppl.table 6 shows the detailed response pattern to the items relating to knowledge, practice, and attitudes about Covid-19 and Covid-19 vaccine. Overall, the majority agreed to some extent that: the vaccine is crucial for public health (62.2% for 'definitely agree', and 28.5% for 'somewhat agree'); Isolation and treatment of Covid-19 is an effective way to reduce the transmission of the virus (88.2% for 'agree'); All vaccines approved to the market are safe (47.5% for 'definitely agree', and 32.4% for 'somewhat agree'). And most people followed that: wearing mask in public places (95.4%); reduce frequency of going out (97.2%); reduce taking public transportation (95.0%); keep social distance (96.5%); wash hands frequently (97.7%). And most of respondents can choose the right options as follows: elderly people, patients with chronic diseases are more likely to develop severe symptoms if they infected with Covid-19 (53.2%); Covid-19 patients without fever cannot infect others (75.0%); Children and adolescent do not need to take measures to prevent Covid-19 (84.6%).

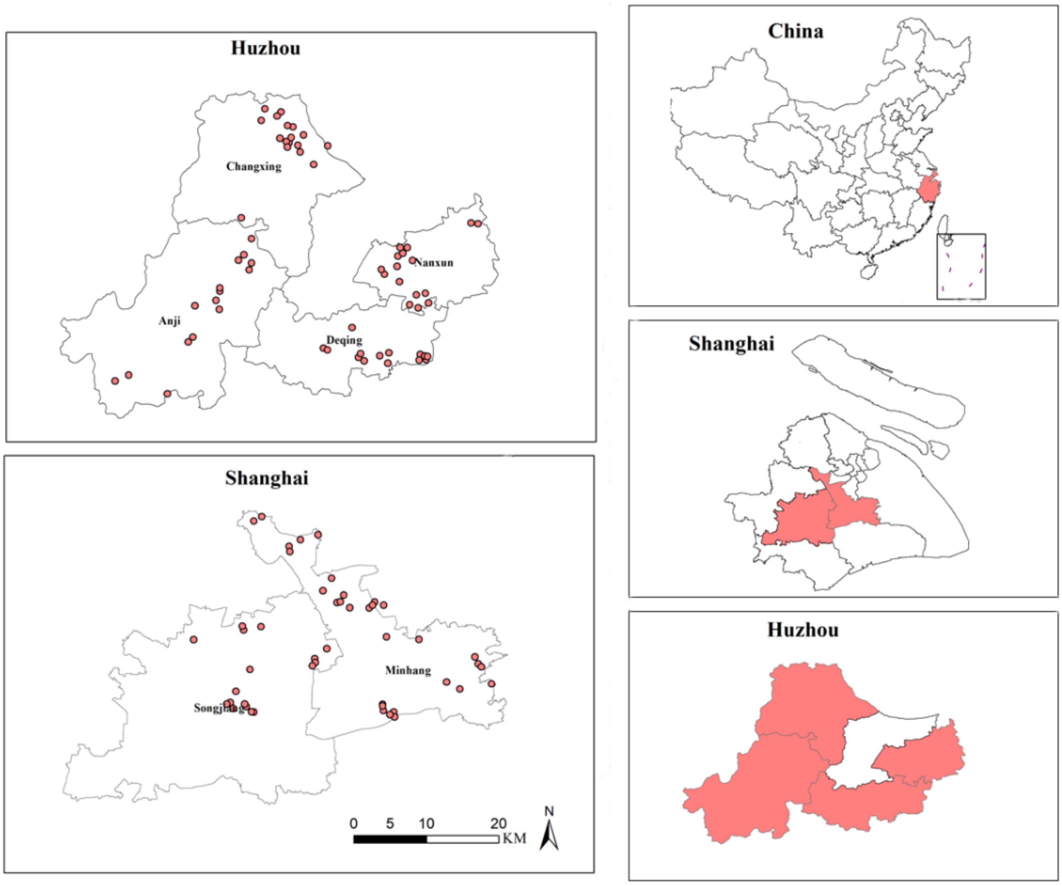

Figure S1 Six districts (counties) sampled and 120 neighborhoods locations

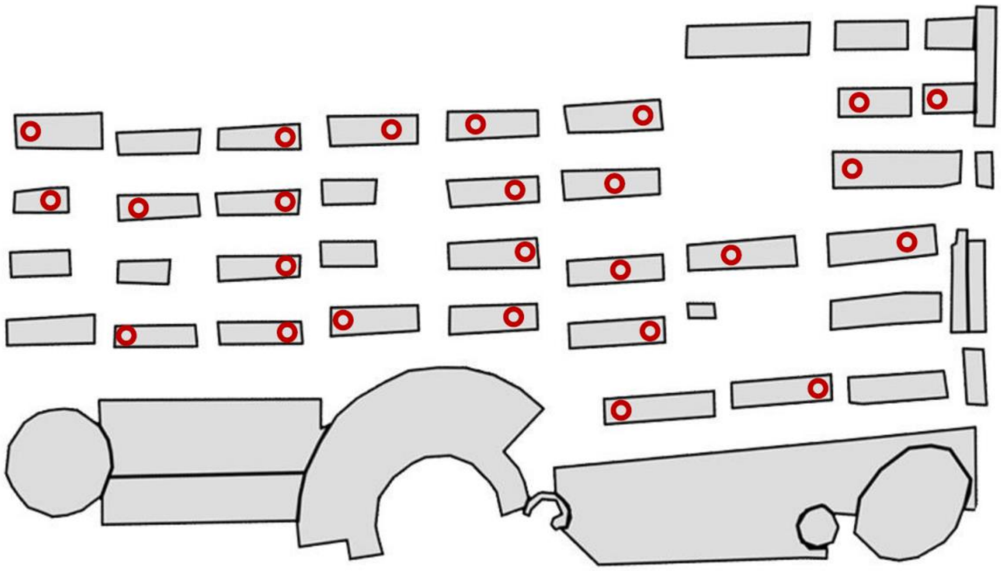

Figure S2. Home locations of participants sampled from one neighborhood for instance. Polygons and open circles represent buildings and home locations, respectively. This figure was built on National Platform for Common Geospatial Information Services (https://www.tianditu.gov.cn/).

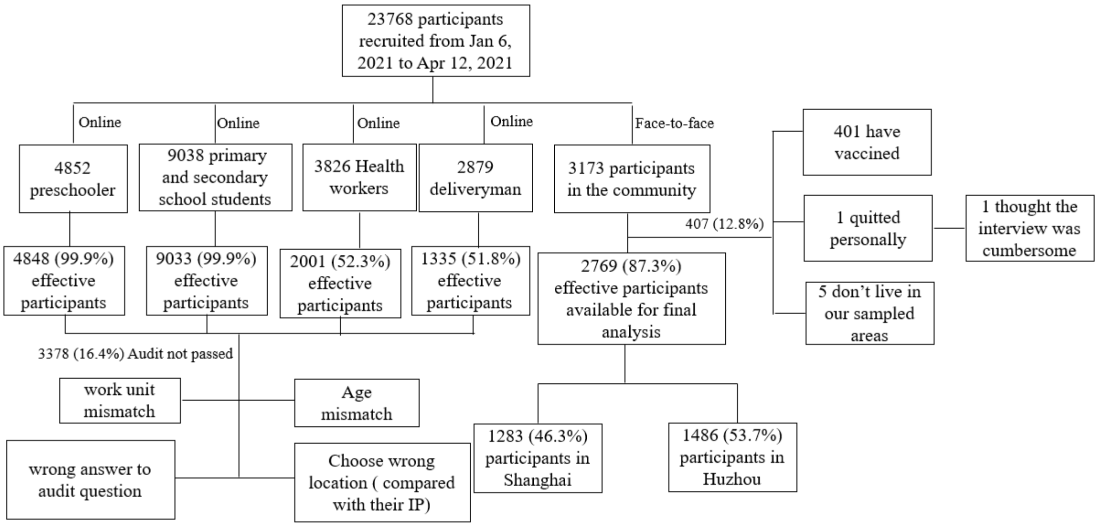

Figure S3. Flow chart from enrollment to analysis for the 23,786 participants

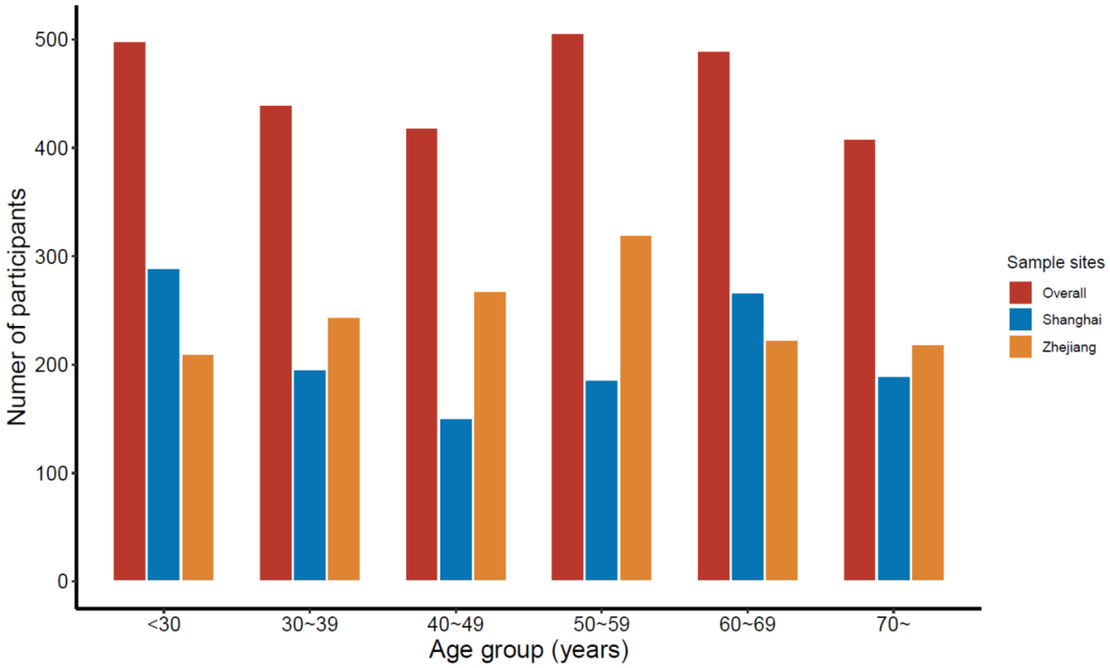

Figure S4 Age distribution of the participants in the community

Table S1. Age distribution of Shanghai and Huzhou population vs. our sample in Shanghai and Huzhou

Age group	<30	30-39	40-49	50-59	60-69	70+
Shanghai (%)	36.06	17.59	15.98	15.32	7.84	7.24
Sample in Shanghai (%)	24.08	15.04	10.44	13.09	17.54	19.80
Zhejiang (%)	18.38	19.19	25.38	17.16	10.60	9.29
Sample in Zhejiang (%)	14.13	16.42	18.03	21.53	15.01	14.74

Table S2 Comparison between effective participants in Shanghai and permanent residents in Shanghai

Characteristics	Population of Shanghai (%) (n=23,019,196)	Participants (%) (n=1283)	*P*[*]
Sex (Male)	11,854,916 (51.50)	583 (45.44)	>0.05
Age group			
[0,30)	8,295,582 (36.04)	296 (24.08)	0.2443
[30,40)	4,049,413 (17.59)	206 (15.04)	
[40,50)	3,678,414 (15.98)	254 (10.44)	
[50,60)	3,526,132 (15.32)	287 (13.09)	
[60,70)	1,803,695 (7.84)	176 (17.54)	
70+	1,665,960 (7.24)	267 (19.80)	

***indicates the results of chi-square test.**

Table S3 Comparison between effective participants in Huzhou and permanent residents in Huzhou

Characteristics	Population of Huzhou (%) (n=2,893,542)	Participants (%) (n=1486)	*P*[*]
Sex (Male)	1,470,472 (50.82)	703 (47.31)	>0.05
Age group			
[18,30)	433481 (18.38)	210 (14.13)	0.2243
[30,40)	452581 (19.19)	244 (16.42)	
[40,50)	598509 (25.38)	268 (18.03)	
[50,60)	404528 (17.16)	320 (21.53)	
[60,70)	249927 (10.60)	223 (15.01)	
70+	219036 (9.29)	219 (14.74)	

***indicates the results of chi-square test.**

Table S4 Comparison between effective participants in our study and residents in China

Characteristics	Population of China (%) (n=1,332,810,869)	Participants (%) (n=2769)	*P*[*]
Sex (Male)	682,329,104 (51.19)	1286 (46.44)	>0.05
Age group			
[18,30)	273185649 (25.86)	605 (21.85)	0.2243

[30,40)	215164162 (20.37)	399 (14.41)
[40,50)	230348517 (21.81)	388 (14.01)
[50,60)	160065645 (15.15)	455 (16.43)
[60,70)	99780564 (9.45)	401 (14.48)
70+	77813876 (7.37)	521 (18.82)

Table S5 Acceptance rate of two vaccine scenarios

	Vaccine A (about 80% effect, domestic)		Vaccine B (about 95% effect, import)	
	**Willingness**	**Unwillingness**	**Willingness**	**Unwillingness**
Overall (%)	75.3	27.4	46.4	53.6
Shanghai (%)	64.9	35.1	40.5	59.5
Zhejiang (%)	84.2	15.8	51.4	48.6

Table S6. Description of community participants.

	Overall
n	2769
fullage (mean (SD))	45.95 (19.29)
Sample sites (%)	
Anji	378 (13.7)
Deqing	351 (12.7)
Minhang	719 (26.0)
Nanxun	402 (14.5)
Songjiang	564 (20.4)
Changxing	355 (12.8)
Sex = female (%)	1483 (53.6)
Region = rural (%)	1469 (53.1)
Marriage = others (%)	557 (20.1)
Ethnic = minority (%)	28 (1.0)
Religion = yes (%)	319 (11.5)
Years of residence (%, years)	
<1	34 (1.2)
1-5	248 (9.0)
6-10	196 (7.1)
≥10	2276 (82.2)
don't know	15 (0.5)
Distance from vaccination site (%, km)	
<1	234 (8.5)
1-2.9	661 (23.9)
3-4.9	525 (19.0)

5-6.9	183 (6.6)
≥7	347 (12.5)
don't know	819 (29.6)
Education (%)	
high	755 (27.3)
low	1472 (53.2)
medium	542 (19.6)
Employment status = Unemployed (%)	792 (28.6)
Been Quarantined = yes (%)	95 (3.4)
Income (%, thousand)	
>10	574 (20.7)
10-30	587 (21.2)
30-50	648 (23.4)
50-100	711 (25.7)
100-200	195 (7.0)
200-300	30 (1.1)
≥300	24 (0.9)
Overseas experience = yes (%)	16 (0.6)
Basic Diseases = Yes (%)	813 (36.9)
Had Covid-19 (%)	
never	2739 (98.9)
confirmed	1 (0.0)
suspected	1 (0.0)
don't know	28 (1.0)
Familiar people been quarantined (%)	
no	2543 (91.8)
yes	130 (4.7)
don't know	96 (3.5)
FrontLines of the epidemic (%)	
No	2245 (81.1)
yes	442 (16.0)
don't know	82 (3.0)
Familiar people on the front lines of the epidemic (%)	
No	1633 (59.0)
yes	903 (32.6)
don't know	233 (8.4)
Familiar people had Covid-19 = yes (%)	19 (0.7)
Health (%)	
poor	54 (2.0)
good	2126 (76.8)
medium	589 (21.3)

Table S7 Knowledge, attitude, and practice about Covid-19 and Covid-19 vaccine

	Overall
n	2769
Older people, people with chronic diseases, are more likely to develop severe Covid-19 (%)	
no	754 (27.2)
yes	1473 (53.2)

don't know	542 (19.6)
Covid-19 patients without fever cannot infect others (%)	
no	2076 (75.0)
yes	370 (13.4)
don't know	323 (11.7)
Children and young adults do not need to take measures to prevent Covid-19 (%)	
no	2343 (84.6)
yes	224 (8.1)
don't know	202 (7.3)
Isolation and treatment of Covid-19 is an effective way to reduce transmission of the virus (%)	
no	128 (4.6)
yes	2442 (88.2)
don't know	199 (7.2)
wearing mask (%)	
no	100 (3.6)
yes	2642 (95.4)
don't know	27 (1.0)
reduce going out (%)	
no	55 (2.0)
yes	2691 (97.2)
don't know	23 (0.8)
reduce taking public transportation (%)	
no	101 (3.6)
yes	2630 (95.0)
don't know	38 (1.4)
keep social distance (%)	
no	60 (2.2)
yes	2672 (96.5)
don't know	37 (1.3)
wash hands frequently (%)	
no	35 (1.3)
yes	2704 (97.7)
don't know	30 (1.1)
Vaccines are very important for the protection of health (%)	
definitely disagree	1 (0.0)
somewhat disagree	25 (0.9)
neutral	135 (4.9)
somewhat agree	790 (28.5)
definitely agree	1722 (62.2)
don't know	96 (3.5)
All vaccines approved to the market are safe (%)	
definitely disagree	10 (0.4)
somewhat disagree	73 (2.6)

neutral	246 (8.9)
somewhat agree	896 (32.4)
definitely agree	1314 (47.5)
don't know	230 (8.3)
