# Supplementary material for: Factors That Impact Acceptance of COVID-19 Vaccination in Different Community-Dwelling Populations in China
Source: Vaccines (Basel). 2022 Jan 8;10(1):91. doi: 10.3390/vaccines10010091 (PMC8779453; doi:10.3390/vaccines10010091)
Supplement: Supplementary file 1 [file vaccines-10-00091-s001.zip › Supplementary material 2.pdf]

vaccines

Supplementary Text 2

The list of schools by region is as follows (The schools in red are the schools that have been selected):

Deqing

High Schools

Deqing High School; Xinshi Vocational High School; Deqing Qiushi High School

Middle Schools

Deqing No.1 Middle School; Deqing No.3 Middle School; Deqing No.5 Middle School; Deqing No.2 Middle School; Deqing No.4 Middle School; Wukang Middle School; Xinshi Town Middle School; Leidian Middle School; Yuyue Middle School; Qianqiu Foreign Language School; Huashengda Foreign Language School; Qiushan central school; Zhongguan Central School; Ganshan Central School; Luoshe Central School; Xiashe Central School; Gouli Central School; Mogan Mountain Central School; Sanhe Central School; Fatou Central School; Mogan Mountain Foreign Language School; Wuyang School; Yucai School; Yuxiu School

Primary Schools

Yifu Primary School; Experimental School; Wukang Yingxi Primary School; Peikang School; Three Bridge Primary School; Shangbai Primary School; Dongfeng Primary School; Qingxi Primary School; Qianyuan Primary School; Xinshi Primary School; Xinshi Central Primary School; Shilin Primary School; Leidian Primary School; Xujiazhuang Primary School; Gaoqiao Primary School; Erdu Primary School; Chunhui Primary School

Kindergartens

Central Kindergarten; Government kindergarten; No. 1 Experimental Kindergarten; No.

2 Experimental Kindergarten; Yuanwang Kindergarten; Dixin Town Kindergarten; Wukang Kindergarten; Shangbai Kindergarten; Sanqiao Kindergarten; Qianqiu Kindergarten; Qianyuan First Kindergarten; Qianyuan Second Kindergarten; Xinshi First Kindergarten; Xinshi Second Kindergarten; Shilin Kindergarten; Zhongguan Kindergarten; Ganshan Kindergarten; Luoshe Kindergarten; Leidian First Kindergarten; Raidian Second Kindergarten; Xujiazhuang Kindergarten; Gaoqiao Kindergarten; Xiashe Kindergarten; Xinan Kindergarten; Mogan Kindergarten; Fatou Kindergarten Sanhe Kindergarten; Qiushan Kindergarten; Jinbeilei Kindergarten; Greentown Qingan Kindergarten; Sunshine Kindergarten; Tashan Kindergarten; Xinsheng Kindergarten Urban Taoyuan Kindergarten; Huilong District Kindergarten; Zhenxing Kindergarten Yuanyuan Kindergarten; Kangle Kindergarten; Lele Kindergarten; Wulong Love Kindergarten; Yidun Education Kindergarten; Jindu Noyazhou Kindergarten

Anji

High Schools

Anji High School; Tianlue Foreign language School; Changshuo High School; Xiaofeng High School; Shangshu Private High School; Anji Zhenmin High School

Middle Schools

Anji Experimental Middle School; Second Middle School; Third Middle School; Nanbeizhuang Middle School; Xiaofeng Middle School; Tianlue Foreign language School; Shangshu Middle School
Gaoyu Middle School.

Primary Schools

Experimental Primary School; Second Primary School; Third Primary School; Fenghuang Mountain Primary School; Tianlue Foreign Language School; Fourth Primary School; Fifth Primary School; Sixth Primary School

Seventh Primary School; Eighth Primary School

Kindergartens

Anxiao Kindergarten; Anxiang Kindergarten; Xinxin Kindergarten; Red, Yellow and Blue Kindergarten; Guoguo Kindergarten; Small Sun Kindergarten; Xin'aiying Kindergarten; Aimi'er Kindergarten; Tangram Kindergarten

Golmud

High Schools

Golmud No.1 Middle School; Golmud No.2 Middle School; Golmud No.7 Middle School; Golmud No.8 Middle School; National Middle School; East Village Yifu School of Guo Towh; Guo Town Central School; Xinhua Middle School; Daxiang middle school; Jinghua School

Middle Schools

Golmud No.3 Middle School; Golmud No.4 Middle School; Golmud No.5 Middle School; Golmud No.6 Middle School; Golmud No.9 Middle School; Golmud No.10 Middle School; Golmud No.11 Middle School; Golmud No.12 Middle School; Golmud No.13 Middle School

Primary Schools

Jinfeng Road Primary School; Experimental Primary School; Zhongshan Road Primary School; Jiangyuan Road Primary School; Zhanqian Road Primary School; Changjiangyuan National Primary School; Yucai Primary School; Xinmiao Boarding School (private); Baoku Village Primary School of Guo Town; New Village Yifu School of Guo Town

Kindergartens

Golmud City Kindergarten; Liuyi Kindergarten; Kunlun Kindergarten; Bayi Kindergarten; Little Sun Kindergarten; Little sun Sinology Museum; Guo Town Central Bilingual Kindergarten; Xinhua Bilingual Kindergarten; Daxiang Kindergarten Airui Art Kindergarten; Little Doctor Kindergarten; Changjiang Yuan National Kindergarten; Little Red Riding Hood Kindergarten (private); Xinlei Kindergarten; Yanhu Kindergarten; Petrochemical base kindergarten; Hedong Kindergarten; Chunhui Kindergarten; Xiangyang Kindergarten; Blue Sky Kindergarten; Minle Kindergarten; Chenguang Kindergarten; Yuanqu Kindergarten

Nanxun

High Schools

Jinxiu Experimental School; Hongda High School; Shanlian High School; Shicong High School

Primary Schools

Xunxi Middle School; Hengjie Middle School

Sanjie School; The Experimental School of the Development Zone; Crystal New Town School; Hefu Middle School; Jiuguan Middle School; Lianshi No. 1 Middle School; Linghu No. 1 Middle School; Linghu No. 3 Middle School; Qianjin Middle School; Shuanglin No. 2 Middle School; Nanxun Middle School; Linghu Middle School; Liancheng Middle School; Shuanglin Middle

Primary Schools

Mayao Primary School; Nanxun Experimental Primary School; Nanxun Affiliated Primary School of Huzhou University; Xiangyang Primary School; Limin Primary School; Hengjie Primary School; Xunxi Campus; Sijin Primary School; Shuanglin Qinglong Primary School; Luofu Primary School; Zhenxi Primary School; Lianshi Primary School; Guoqiang School; Hongtang Primary School; Hualin Primary School Linghu Experimental Primary School; Linghu No.3 Primary School; Linghu No.5

Primary School; Quansheng Primary School; Changchao Primary School; Chongzhao Primary School; Jiuguan Prinwy School; Qianjin Primary School; Hanshan Primary School

Kindergartens

Mayao Kindergarten; Hongda Kindergarten; Nanxun Experimental Kindergarten; Nanxun Experimental Kindergarten of Chaoyang Park; Nanxun Town Kindergarten; Nanxun Town Kindergarten Convenience Road Branch Park; Nanxun New District Kindergarten; Jinxiang Lake Experimental Kindergarten; Yansi Kindergarten; Xiucheng Kindergarten; Golden Apple Kindergarten; Xiangye Bay Kindergarten; Huafu Art Kindergarten; Moon Bay Golden Cradle Kindergarten; Hengjie Kindergarten; South City Kindergarten; Sanchang Kindergarten; Nanxun Town Youth Tutor Centre; Dongqian Kindergarten; Noah Boat Kindergarten; Tangbei Huatian Kindergarten; Nankai Experimental Kindergarten; Jiuguan Bebelle Kindergarten; Jiuguan Town Times Kindergarten; Jiuguan Town Central Kindergarten; Lianshi Town Central Kindergarten; Hongtang Branch Park; Lianshi Town Rube Kindergarten; Hualin Kindergarten; Lianshi Town Jingyue Bay Kindergarten; Lianshi Jianxin Branch Park; Linghu Town Kindergarten; Xixi Branch Park; Xiaang Branch Park; Linghu New Century Kindergarten; Shuanglin Town Central Kindergarten; Shuanglin Boya Kindergarten; Shuanglin Love Kindergarten; West Town Branch Park; Hefu Town Central Kindergarten; Chongzhao Kindergarten; Changchao Kindergarten; Shanlian Town Central Kindergarten; Hanshan Kindergarten; Qianjin Town Central Kindergarten; Shicong Town Central Kindergarten

Songjiang

Kindergarten

Shanghai Songjiang District Happy Kindergarten; Shanghai Hurd Kindergarten

Shanghai Songjiang District Blue Moon Kindergarten; Shanghai Songjiang District Happy Childhood Early Education Center (closed, not cancelled); Shanghai Songjiang

District Bosi Zhiying Kindergarten Co., Ltd; Shanghai Songjiang District Private Rainbow Kindergarten; Shanghai Songjiang District Xinxin Baby Kindergarten; Shanghai Songjiang District Yuxiu Kindergarten; Shanghai Songjiang District Yushu Kindergarten; Shanghai Songjiang Eddie Kindergarten; Shanghai Xiyuan Foreign Language Kindergarten; Shanghai Songjiang District Xinjiyuan Lilac Kindergarten; Shanghai Xuexing Green Orange Kindergarten; Songjiang Fangsong Nursery, Shanghai; Shanghai Harmony Kindergarten; Shanghai Golden Baby Parent-Child Early Education Center (closed, not cancelled); Shanghai Songjiang District Quyaya Early Education Center; Shanghai Songjiang District Lezhi Kindergarten; Shanghai Songjiang Blue Snail Kindergarten; Shanghai Songjiang District Xinye Kindergarten (closed, not cancelled); Shanghai Songjiang District Chunyang Kindergarten; Shanghai Songjiang District Yulei Kindergarten; Shanghai Songjiang District Leying Kindergarten; Shanghai Songjiang District Preschool Tiandi BiHuali Bilingual Kindergarten; Shanghai Songjiang District Oriental Lan Valley Enlightenment Kindergarten; Shanghai Lejing Sports Kindergarten; Shanghai Songjiang District Chunshen Kindergarten; Shanghai Songjiang Rongsheng Parent-Child Park; Shanghai Songjiang District Fiso Kindergarten; Shanghai Songjiang District Yuanzhi Kindergarten; Shanghai Songjiang District Hongtongtong Kindergarten; Shanghai Songjiang District Miaosan Road Kindergarten; Shanghai Songjiang District Xinqiao Happy Kindergarten; Shanghai Songjiang District YueMeichuang Kindergarten; Shanghai Songjiang District Xinran Kindergarten; Shanghai Songjiang District Yuehuipo Kindergarten; Shanghai Songjiang District New Blue Kindergarten; Shanghai Songjiang District Lexi Manor Kindergarten; Shanghai Songjiang District Jinhuan Kindergarten; Shanghai Songjiang District Jiadebao Kindergarten; Shanghai Songjiang District Yiqi Kindergarten Co., Ltd.; Shanghai Songjiang District Ivy Greenlading Kindergarten; Shanghai Songjiang District New Wenhua Kindergarten; Shanghai Songjiang District Xingang East Kindergarten (closed, not cancelled); Shanghai Songjiang Jiuzhou Kindergarten; Shanghai Songjiang District Boya Kindergarten (closed, not cancelled); Shanghai Golden Childhood Art Kindergarten

Shanghai Songjiang District Chunhui Bilingual Art Kindergarten; Shanghai Songjiang Pearl of the Palm Experimental Kindergarten; Shanghai Songjiang Jingjiang Kindergarten; Shanghai Songjiang District Dile Kindergarten; Shanghai Songjiang District Qizhi Kindergarten; Shanghai Songjiang District Yibei Kindergarten; Shanghai Songjiang District Huizhixing Kindergarten; Shanghai Songjiang District Enlightenment Kindergarten; Shanghai Songjiang District Sheshan Ivy Kindergarten Shanghai Songjiang District Huiming Kindergarten; Shanghai Songjiang District Shize Kindergarten; Shanghai Songjiang District Happy World Kindergarten; Shanghai Modern Pearl Kindergarten; Shanghai Songjiang District Jiahua Art Kindergarten; Shanghai Songjiang District Xingchen Kindergarten; Shanghai Songjiang District Hanying Kindergarten; Shanghai Songjiang District Angel Bell Kindergarten; Shanghai Songjiang District Kaiqi Kindergarten; Shanghai Songjiang District Jiuhong Kindergarten; Shanghai Songjiang District Xinxing Baby Early Education Center; Shanghai Songjiang District Mengzhen Kindergarten (closed, not cancelled); Shanghai Songjiang District Qixing Kindergarten; Linsong Kindergarten Affiliated to Shanghai Songjiang District International Education; Shanghai Songjiang District Disheng Kindergarten; Shanghai Songjiang District Aisitan Kindergarten; Shanghai Songjiang District Moon Boat Bilingual Kindergarten; Shanghai Songjiang District Creative Kindergarten (closed down, not cancelled); Shanghai Songjiang District Laiyin Jiahua Kindergarten; Shanghai Songjiang District Green Lanting Bilingual Kindergarten; Shanghai Songjiang District Hand in Hand Kindergarten; Shanghai Songjiang District Duxiang Kindergarten; Shanghai Songjiang District Jinhong Kindergarten; Shanghai Songjiang District Grite Kindergarten; Shanghai Songjiang District Love Kindergarten Shanghai Songjiang District Integrity Kindergarten; Shanghai Songjiang District Flying Kindergarten; Shanghai Songjiang District Jinbei Art Kindergarten; Shanghai Songjiang District Wuzhou Kindergarten; Shanghai Songjiang District Yongxiang Kindergarten; Shanghai Songjiang District Zhongqing Reed Kindergarten; Shanghai Songjiang District Yiyoufan Kindergarten; Shanghai Songjiang District ailang Kindergarten; Shanghai Songjiang Xiangdong Kindergarten; Shanghai Songjiang

Private Oriental Kindergarten; Shanghai Songjiang District Xinnan Kindergarten; Shanghai Songjiang District Small Grape Kindergarten

Minhang

High School

Shanghai Gumei High School; Shanghai Private Liaoyuan Bilingual Senior High School; Shanghai Wenlai Middle School; Shanghai Jinhui Senior High School; Shanghai Union Bilingual High School; Shanghai Minhang District Nord Anda Bilingual School; Minhang Foreign Language Middle School of Shanghai International Studies University; Shanghai Jiaotong University Affiliated Middle School Minhang Branch; Shanghai Private Wenqi Middle School; Shanghai Minhang Third Middle School; Shanghai Minhang Middle School; Minhang High School of Science and Technology Affiliated to East China University of Science and Technology; Shanghai Minhang District Private Dehong School; Shanghai Minhang District Union Bilingual Education Science School; Shanghai Second Middle School Meilong Campus; Pujiang High School Affiliated to Shanghai University of Traditional Chinese Medicine; Minhang Branch of High School Affiliated to Shanghai Normal University; Shanghai Xiangming Middle School; Shanghai Qibao Dwight High School; Shanghai Qibao Middle School; Shanghai Minhang District School of Education Affiliated Middle School; Shanghai Xinzhuang Middle School; The Second High School Affiliated to East China Normal University Zizhu Campus; Shanghai Minhang Garden High School affiliated to Beijing Foreign Studies University; Shanghai Qibao Middle School Affiliated Xindu Experimental Middle School; Shanghai Star River Bilingual School; Shanghai Qunyi Vocational Technical School

Middle School

Shanghai Minhang District Pujiang First Middle School; Shanghai Minhang Vanke Bilingual School; Shanghai Hanghua Middle School; Shanghai Private Fudan Vanke Experimental school; Shanghai Shangbao Middle School; Shanghai Minhang District

Qibao Second Middle School; Shanghai Minhang District Qibao Second Middle School North Campus; Shanghai Minhang District Qibao Third Middle School; Shanghai Minhang District Qiyin School; Shanghai Qibao Experimental Middle School; Shanghai Wenlai Middle School Middle School; Shanghai Normal University Kangcheng Experimental School, Shenbei Road Teaching Site; Shanghai Union Private Bilingual Shangyin School; Shanghai Minhang District Star School; Shanghai Minhang District Youth Amateur Sports School; Shanghai Minhang District Xinsong Middle School; Shanghai Minhang District Xinsong Middle School Chunshen Campus; Shanghai Minhang District Shuiqing Road Xinsong Middle School; Shanghai Xincheng School; Shanghai Xinguang School; Minhang Experimental Middle School Affiliated to Shanghai Teacher Training Center; ShangNormal University Kangcheng Experimental School; The Second Affiliated Middle School of East China Normal University Affiliated Junior High School; Shanghai Zizhu Bilingual School of the Second High School Affiliated to East China Normal University; Shanghai Minhang District College of Education Affiliated Youai Experimental Middle School; Shanghai Wujing Middle School; Shanghai Wenlai Experimental School; Shanghai Minhang District Beiqiao Middle School; Shanghai Minhang District Junlian School; Shanghai Minhang District Tiantian Foreign Language Middle School; Shanghai Minhang District Zhuanqiao Middle School; Shanghai Qibao Middle School Affiliated Xindu Experimental Middle School; Shanghai Star River Bilingual School; Shanghai Southwest Engineering School

Primary School

Minhang District Pingyang Primary School; Shanghai Gumei School

Shanghai Liaoyuan Bilingual School; Shanghai Wanyuan City Union Bilingual School; Shanghai Minhang District Pingnan Primary School; Shanghai Minhang District Pingnan Primary School Pingji Campus; Shanghai Rixin Experimental Primary School; Minhang District Hongqiao Central Primary School; Minhang District Longbai First Primary School; Shanghai Jinhui Experimental School; Shanghai Private Union

Bilingual School; Shanghai Minhang District Huahong Primary School; Shanghai Minhang District Huaboli Xingxing Primary School (Huacao); Shanghai Minhang District Huboli Xingxing Primary School Jiwang Campus;Shanghai Minhang District Private Meigao Bilingual School; Shanghai Minhang District Nord Anda Bilingual School; Shanghai Minhang District Huacao School; Shanghai Minhang District Jiwang School; Shanghai Minhang District Zhuzhai School; Experimental Primary School Affiliated to Shanghai Jiao Tong University; Shanghai Minhang District Office Shuangjiang Primary School; Shanghai Minhang Bijiang Primary School; Shanghai Minhang Fushan Experimental School; Shanghai Minhang Hebei Primary School; Shanghai Minhang Huaping Primary School; Shanghai Minhang Jiangchuan Road Primary School; North Campus of Shanghai Minhang Jiangchuan Road Primary School; Shanghai Minhang Kunyang Primary School; Shanghai Minhang District Qizhi School; Shanghai Minhang District Steam Turbine Technology Experimental Primary School; Shanghai Minhang District Central Primary School; Shanghai Minhang District Private Dehong School; Shanghai Minhang District Maqiao Primary School Shanghai Maqiao Qiangshu School; Shanghai Minhang District Maqiao Experimental Primary School; Shanghai Minhang District Maqiao Wenlai Foreign Language Primary School; Minhang Primary Experimental School Affiliated to Fudan University ; Meilong Central Primary School; Minhang District Luoyang Primary School; Minhang District Experimental Primary School Chuncheng Campus; Minhang District Experimental Primary School Jingcheng Campus; Minhang District Experimental Primary School WanDing Campus; Shanghai Minhang District Gumei Campus of Private Hongmei Second Primary School; Shanghai Minhang District Hong Mei Primary School; Shanghai Minhang District Private Hong Mei Primary School Qiangwei Campus; Shanghai Minhang District Private Hongmei Primary School Zhuxing Campus; Shanghai Minhang District Private Yumiao Primary school; Shanghai Minhang District Caohang Primary School; Minhang Qiangwei Primary School Affiliated to Shanghai University of Traditional Chinese Medicine; Jingcheng Campus of Minhang Qiangwei Primary School Affiliated to Shanghai University of

Traditional Chinese Medicine; Shanghai Boskey Foreign Language School; Shanghai Minhang District Wenhui School; Shanghai Minhang District Wenxin School; Shanghai Shiwai Education Affiliated Pujiang Foreign Language School; Shanghai Minhang District Pujiang Huixiu Primary School; Shanghai Minhang District Puhui Primary School; Shanghai Minhang District Puhui Second Primary School; Shanghai Minhang District Putao Road Campus Pujiang Second Primary School; Shanghai Minhang District Pujiang Third Primary School; Minhang Affiliated School of Shanghai Theatre Academy; Minhang Third Primary School Affiliated to Shanghai Normal University; Shanghai Minhang District Huangpu First Center Shibo Primary School ; Shanghai Minhang District Wenhe Primary School; Huangpu First Center Pujiang First Primary School; Minhang District Hanghua Second Primary School; Minhang District Liming Primary School; Minhang District Qibao Town Mingqiang Primary School; Minhang District Qibao Town Mingqiang Primary School West Campus; Shanghai Minhang District Private Zhenxing Primary School; Shanghai Minhang Vanke Bilingual School; Shanghai Private Fudan Vanke Experimental school; Shanghai Minhang District Qibao Mingqiang Second Primary School; Shanghai Minhang District Qiyin School; Shanghai Qibao Experimental Primary School; Shanghai Qibao Experimental Primary School, Greater Shanghai Campus; Shanghai Qibao Foreign Language Primary School; Minhang District Garden School; Minhang District Xinzhuang Town Primary School; Minhang District Xinzhuang Town Primary School South Campus; Minhang District Experimental Primary School; Minhang District Xinmei Primary School; Shanghai Union Private Bilingual Shangyin School; Shanghai Minhang District Star School; Shanghai Minhang District Xinsong Primary School; Shanghai Xincheng School; Shanghai Shenguang School Shanhua Campus; ShangNormal University Kangcheng Experimental School; Zizhu Primary School Affiliated to East China Normal University; Minhang Yongde Experimental Primary School of East China Normal University; Shanghai Zizhu Bilingual School of the Second High School Affiliated to East China Normal University; Shanghai Minhang District Private Tangwan Primary School; Shanghai Minhang District Jingdong

Primary School; Shanghai Minhang District Wujing Experimental Primary School; Minhang District Hanghua First Primary School; Shanghai Wenlai Experimental School; Minhang District Beiqiao Central Primary School; Minhang District Zhuanqiao Central Primary School; Shanghai Minhang District private Huaxing Primary School; Shanghai Minhang District private Silver Star School; Shanghai Minhang District Junlian School Primary School; Shanghai Minhang District Shenxin Primary School Shanghai Minhang District Tianyuan Second Foreign Language Experimental Primary School; Minhang District, Shanghai Tiantian Foreign Language Experimental Primary School; Shanghai Minhang District Jindu Campus of Tianyuan Experimental Foreign Language Primary School; Shanghai Minhang District Xindu Primary School; Shanghai Star River Bilingual School

Kindergarten

Meilong Central Kindergarten; South Branch of Meilong Central Kindergarten,The Second Village Branch of Meilong Central Kindergarten; JingCheng Branch of Chunshen Jingcheng Kindergarten; Happy Branch of JingCheng Branch of Chunshen Jingcheng Kindergarten; Chunshen Branch of Chunshen Jingcheng Kindergarten; Jingcai Square Kindergarten; Jinghua Fang Kindergarten; Luoyang Riverside Kindergarten; Luoyang Branch of Luoyang Riverside Kindergarten; Jinglian Branch of Cao Hang Center Kindergarten; Qiangwei Branch of Cao Hang Center Kindergarten Jindu Kindergarten; Yintai Branch of Jindu kindergarten; Wangzu Yuan Kindergarten Xiao Miao Kindergarten; Yingtaozi Kindergarten; Century Garden Yingtaozi Kindergarten; Good Time Golden Thumb Kindergarten; Union Luoyang Kindergarten Golden Peacock Kindergarten; Meilong Private Xingnan Kindergarten; Ugly Duckling Kindergarten; Meilong Private Century Kindergarten; Minhang District East China Normal University Knowledge Order Experimental Kindergarten; Jinggu Eryou Kindergarten; First Branch of Jinggu Eryou Kindergarten; Second Branch of Jinggu Eryou Kindergarten; Minhang District Jinggu First Kindergarten; Ruili Branch of Jinggu First Kindergarten; Shanghai Minhang District Kunyang Kindergarten; Jinying

Branch of Minhang District Kunyang Kindergarten; Miaomiao Branch of Minhang Kunyang Kindergarten; Jinbang Branch of Heqing Kindergarten; Lanping Branch of Heqing Kindergarten; Nordic Branch of Heqing Kindergarten; Shanghai Jiaotong University Minhang Kindergarten; Dongchuan Branch of Shanghai Jiao Tong University Minhang Kindergarten; Minhang District Anning Road Kindergarten

Minhang District First Kindergarten; Changxin Branch of Minhang District First Kindergarten; Shanghai Minhang District Private Jiangchuan Huining Road Kindergarten; Xinhua Wansu Kindergarten; Union Sunshine Kindergarten; Minhang District Xinhua Kindergarten; Qiandai Branch of Minhang District Fourth Kindergarten; Jinbang Branch of Minhang District Fourth Kindergarten; Pujiang Branch of Minhang District Fourth Kindergarten; Ronghua Branch of Minhang District Fourth Kindergarten; Xinmin Branch of Minhang District Fourth Kindergarten; Minhang District Shanghai Small Harvard Haodifang Kindergarten; Minhang District Pujiang Town Second Kindergarten; Jinshuo Branch of Minhang District Pujiang Town Second Kindergarten; Minhang District Pujiang Town Third Kindergarten; Purun Branch of Minhang District Pujiang Town Third Kindergarten; Minhang District Puhang Kindergarten; Zhongcheng Branch Minhang District Puhang Kindergarten; Minhang District Pujiang Baodi Kindergarten; Ruihe Branch Minhang District Pujiang Baodi Kindergarten; Shuiyu Branch Minhang District Pujiang Baodi Kindergarten

Minhang District Pulian Kindergarten; Jiaxing Branch Minhang District Pulian Kindergarten; Minhang District Pujiang Zhahang Road Kindergarten; Minhang District Pujiang Ruihe City Kindergarten; Minhang District Lehai Kindergarten; Pujiang Star Kindergarten; Minhang District Private Pujiang Sumin Kindergarten; Minhang District Private Wenbo Kindergarten; Zhaolou Kindergarten; Hailida Chunshen Kindergarten; Jia Jia Xintiandi Kindergarten; Jiachen Aiyi Kindergarten; Jiangnan Haisu Kindergarten; Jinmin Lianpo Kindergarten; Kim Beibei Kindergarten; Li 'an Shiyan Kindergarten; Minhang District Bowen Kindergarten; Minhang District Jia Jia Central Kindergarten; Liandong Branch of Minhang District Jiajia Central Kindergarten; Minhang District Xinsong Second Kindergarten; Baochun Branch of Minhang District

Xinsong Second Kindergarten; Minhang Xinsong Kindergarten; Xinjiyuan Branch of Minhang District Xinsong Kindergarten; Sijiyuan Branch of Minhang District Xinsong Kindergarten; Minhang District Xinzhuang Third Kindergarten; Minhang District Shuiqing Road Kindergarten; Minhang District Shuiqing Road Kindergarten Lihua Department; Lvyin Branch of Minhang District Shuiqing Road Kindergarten; Minhang District Shui Qing Road Kindergarten Mingdu Department; Minhang Early Education Center; Minhang District Yilin Kindergarten; Xincheng Haisu Kindergarten; Minhang Children City Yuetting Kindergarten; Minhang District Kangcheng Kindergarten ; Minhang District Green World Shiyan Kindergarten; Minhang District Victoria Kindergarten; Minhang District Victoria Gumeiyou Kindergarten; Qibao Branch of Minhang District Victoria Kindergarten; Xinsong Xiaomiao Kindergarten; Xinzhuang Second Kindergarten Neighborhood Department; Xinzhuang Second Kindergarten Xinzhong Department; Minhang District Xinzhuang Second Kindergarten Qingxin Department; Xinzhuang Kindergarten Qinchun Garden Department; Xinzhuang Kindergarten Zhongchun Garden Department; Minhang District Xinzhuang Kindergarten Pingyang Garden Department; Shengtao Shayou Kindergarten; Century Angli Kindergarten; Xinmei Kindergarten; Xinguang Kindergarten; Xiuwen Kindergarten; Minhang District New Century Hongxin Kindergarten; Zhudi Central Kindergarten; Jiuyun Branch of Zhuzhai Central Kindergarten; Huacao Town Central Kindergarten; Jiwang Kindergarten; Yinxing Branch of Jiwang Kindergarten; Beishen Branch of Golden Kindergarten; Shenchang Branch of Golden Kindergarten; Morgan Henry Kindergarten; Hope Kindergarten; Dream Dora Kindergarten; Hongwei Kindergarten; Hanglong Kindergarten; Oufeng Branch of Gumei Central Kindergarten; Pingji Branch of Gumei Central Kindergarten; Pingnan Branch of Gumei Central Kindergarten; Wanyuan City Kindergarten; Yujing Branch of Wanyuan City Kindergarten; Minhang District New Times Kindergarten; Minhang District Ladder Jimei Kindergarten; Minhang District Small Tadpole Music Kindergarten; Jiachen Jinbaobei Kindergarten; Minhang Spring Light Art Shiyan Kindergarten; Minhang Oriental Cambridge Kindergarten; Gumei Donglan Road Kindergarten; Gumei

Sunshine Kindergarten; Gumei Sunshine Shifang Road Kindergarten; He Luting Kindergarten; Minhang District Longming Road Kindergarten; Hongxin Branch of Longming Road Kindergarten; Mengshi Blue Kindergarten; Minhang Technology Kindergarten; Pingji Sijiefang Kindergarten; Minhang District Yidun Huizhi Jinhui Haoting Kindergarten; Minhang District Hongqiao Central Kindergarten; Gubei Branch of Minhang District Hongqiao Central Kindergarten; Longbai Second Kindergarten; Longbai Second Kindergarten Branch; Honglu Kindergarten; Jingting Branch of Honglu Kindergarten; Minhang District Longbai First Kindergarten; Minhang District Longbai First Kindergarten Branch; Longbai Rainforest Kindergarten Minhang District Xiaolongyu Kindergarten; Longbai Xijiao Kindergarten; Haifu Hongqiao Kindergarten; Minhang District Love Green Kindergarten; Jinhui Shiyan Kindergarten; Union Shiyan Kindergarten; Minhang District Sanzhisan Gubei Kindergarten; Oudun Splendid Kindergarten; Huchuan Kindergarten; Maqiao Fuzhuo Kindergarten; Maqiao Shiyan Kindergarten; Maqiao Central Kindergarten; Fujie Branch of Maqiao Fujie Kindergarten; Yinkang Branch of Maqiao Fujie Kindergarten Maqiao Qiying Kindergarten; Maqiao Yuanxiang Kindergarten; Minhang District Future Baby Kindergarten; Minhang District Pujiang Town First Kindergarten; Lotus Pond World Expo Kindergarten; Lotus Pond World Expo Kindergarten Branch ; Minhang District Wuyi Kindergarten; Shangde Kunting Kindergarten; Zhonghe Kindergarten; Minhang District Jade Kindergarten; Minhang District Yipin Mancheng Kindergarten; Minhang Union Haifu Kindergarten; Golden Sunshine Expo Kindergarten; Zhongfu Club Pujiang Kindergarten; Qibao Central Kindergarten; Jiabao Branch of Qibao Central Kindergarten; Diecai Branch of Qibao Central Kindergarten Laiyin Branch of Qibao Central Kindergarten; Wantai Branch of Qibao Central Kindergarten; Guanghai Central Kindergarten; Xingchen Kindergarten; Golden Sun Branch of Xingchen Kindergarten; Chunxin Kindergarten; Qibao Shiyan Kindergarten Little Star Kindergarten; Qiying Kindergarten; Qiying Maosheng Kindergarten; New Century Jing 'an Xincheng Kindergarten; Heqin Aizhen Kindergarten; Havers Kindergarten; Vanke Yili Sha Bai Kindergarten; Huangdu Kindergarten; Bicheng

Lianming Kindergarten; Hanghua Second Kindergarten; Hanghua Second Kindergarten Branch; Boai Kindergarten; Xiaohongmao Art Kindergarten; Feier Kindergarten; Hanghua Sanyou; Jingding Kindergarten; Vanke Kindergarten; Alice Kindergarten; Fenghua Branch of Minhang District Wujing First Kindergarten; Yongde Branch of Minhang District Wujing First Kindergarten; Jianchuan Branch of Minhang District Wujing First Kindergarten; Minhang District Wujing Second Kindergarten; Minhang District Private Wujing Beibei Kindergarten; Minhang District Private Wujing Flower Kindergarten; Minhang District Private Wujing Nantang Kindergarten Tangwan Central Kindergarten; Minhang China Normal University Yongde Shiyanl Kindergarten; Shangde Branch of Minhang China Normal University Yongde Shiyanl Kindergarten; Wujing Third Kindergarten; Zizhu Kindergarten affiliated to East China Normal University; Minhang District Private Wujing Beier Kindergarten; Minhang Aibo Guoguo Kindergarten; Minhang Aibo Kindergarten; Hualei Branch of Aibo Kindergarten; Xinhong Private Little Doctor Kindergarten; Liu Shikun Music Art Kindergarten; Minhang District Bo 'ai Manting Fang Kindergarten; Minhang District Ivy Kindergarten; Minhang District Dudu Yage Kindergarten; Minhang District Dudu Kindergarten; Minhang District Ha Ha Kindergarten; Minhang District Jinming Kindergarten; Minhang Junlian Kindergarten; Chundu Branch of Minhang District; Minhang District Private Lu Binghua Kindergarten; Minhang District Private New Spring Kindergarten; Minhang District Zhuanqiao Jinsheng Kindergarten; Minhang District Private Zhuanqiao Candlelight Kindergarten; Minhang District Nancy Kindergarten; Minhang District Tianheng Mingdu Kindergarten; Minhang District Xindu Kindergarten; Minhang District Zhuanqiao Town First Kindergarten; Minhang District Zhuanqiao Town First Kindergarten Branch; Minhang District Zhuanqiao Town Riyue Huating Kindergarten; Minhang District Zhuanqiao Town Tianyuan City Kindergarten; Yinsan Minhang District Zhuanqiao Town Tianyuan City Kindergarten; Minhang District Zhuanqiao Town Zhuanxi Kindergarten; Minhang Private Montessori Kindergarten; Fudi Branch of Shanghai Normal University Minhang Shiyan Kindergarten; Taixiang Branch of Shanghai Normal University Minhang Shiyan

Kindergarten; Rongshun Branch of Minhang District Zhuanqiao Town Kindergarten ; Fansheng Branch of Minhang District Zhuanqiao Town Kindergarten; Zimin Branch of Minhang District Zhuanqiao Town Kindergarten; Maqiao Fuguo Kindergarten; Minhang Shiyan Kindergarten; The Welfare Club Nursery

Changxing

High School

The Taihu Lake High School; Changxing Fifth High School; Changxing Seventh High School; Changxing Eighth High School; Educational Practice Centre; Changxing Cultivation School; Jinling High School; Huasheng High School

Spring school; High school of Art; ChangXing Third High School; ChangXing Fourth High School

Middle school

Changxing middle school; Shiukou Middle School; Lijiaxiang Town Middle School; Hongqiao Yifu Middle School; Jiapu Middle school; Shiyan Middle School; Longshan Middle School; Si'an middle School; Gucheng middle School; Zhi City middle School

Houyang Middle School

Primary School

Lijiaxiang Primary School; Heping Middle school; Heping Primary school; Wushan Primary School; Hongxing Bridge Middle School; Hongxing Bridge Primary School

Litang Primary School; Lvshan Middle School; Lv Shan Primary School; Baixian Central Primary School; Huaikan Central Primary School; Meishan Middle School; Meishan Primary School; Xiaopu Middle School; Xiaopu Primary School; Si'an Primary School; Xianshan Primary School; Chang chao Pimary School; Guandi Primary School; Erjieling Primary School; Lincheng Middle School; Lincheng Primary School; Tianping Primary School; Hongqiao Town Central Primary School; Tuying Primary school; Shuikou Primary School; Jiapu Primary school; Dingjiaqiao Central

Primary School; Longbridge Primary School; Taihu Street Central Primary School; Baoqiao Primary school; Shiyan Primary School; The second Shiyan Primary School; Changxing First Primary School; Changxing Sixth Primary School; Changxing Second Primary School

Kindergarten

Jiguan Kindergarten; Shiyan Kindergarten; County Central Kindergarten; Longshan Kindergarten; Shuimu Huadu Kindergarten; Zhangjiacun Garden Kindergarten; Zhicheng Kindergarten; Taihu Street Kindergarten; Development Zone Kindergarten; Longshan Street Central Kindergarten; Changqiao Kindergarten; Baoqiao Kindergarten; Wushan Kindergarten; Heping Kindergarten; Hongxing Bridge Kindergarten; Lvshan Kindergarten; Lijiaxiang Kindergarten; Hongqiao Kindergarten; Tuying Kindergarten; Jiapu Kindergarten; Shuikou Kindergarten; Erjieling Kindergarten; Sian Kindergarten; Lincheng Kindergarten; Xiaopu Kindergarten; Baixian Kindergarten; Huaikan Kindergarten; Meishan Kindergarten; The Blue Sky School
